# Evidence for a compensatory relationship between left- and right-lateralized brain networks

**DOI:** 10.1162/imag_a_00437

**Published:** 2025-01-29

**Authors:** Madeline Peterson, Rodrigo M. Braga, Dorothea L. Floris, Jared A. Nielsen

**Affiliations:** Department of Psychology, Brigham Young University, Provo, UT, United States; Department of Neurology, Northwestern University Feinberg School of Medicine, Chicago, IL, United States; Methods of Plasticity Research, Department of Psychology, University of Zurich, Zurich, Switzerland; Department of Cognitive Neuroscience, Donders Institute for Brain, Cognition and Behaviour, Radboud University Nijmegen Medical Center, Nijmegen, The Netherlands; Neuroscience Center, Brigham Young University, Provo, UT, United States

**Keywords:** lateralization, asymmetry, brain networks, fMRI, language, attention

## Abstract

The two hemispheres of the human brain are functionally asymmetric. At the network level, the language network exhibits left-hemisphere lateralization. While this asymmetry is widely replicated, the extent to which other functional networks demonstrate lateralization remains a subject of investigation. Additionally, it is unknown how the lateralization of one functional network may affect the lateralization of other networks within individuals. We quantified lateralization for each of 17 networks by computing the relative surface area on the left and right cerebral hemispheres. After examining the ecological, convergent, and external validity and test–retest reliability of this surface area-based measure of lateralization, we addressed two hypotheses across multiple datasets (Human Connectome Project = 553, Human Connectome Project-Development = 343, Natural Scenes Dataset = 8). First, we hypothesized that networks associated with language, visuospatial attention, and executive control would show the greatest lateralization. Second, we hypothesized that relationships between lateralized networks would follow a dependent relationship such that greater left lateralization of a network would be associated with greater right lateralization of a different network within individuals, and that this pattern would be systematic across individuals. A language network was among the three networks identified as being significantly left lateralized, and attention and executive control networks were among the five networks identified as being significantly right lateralized. Next, correlation matrices, an exploratory factor analysis, and confirmatory factor analyses were used to test the second hypothesis and examine the organization of lateralized networks. We found general support for a dependent relationship between highly left- and right-lateralized networks, meaning that across subjects, greater left lateralization of a given network (such as a language network) was linked to greater right lateralization of another network (such as a ventral attention/salience network) and vice versa. These results further our understanding of brain organization at the macro-scale network level in individuals, carrying specific relevance for neurodevelopmental conditions characterized by disruptions in lateralization such as autism and schizophrenia.

## 1 Introduction

Observations of the human brain have revealed significant differences in the gross anatomical morphometry between the two hemispheres (for review, seeToga & Thompson, 2003). These structural asymmetries are accompanied by functional asymmetries, most notably for language specialization. Famously, Paul Broca localized language specialization to the left hemisphere subsequent to identifying a lesion in the left inferior frontal gyrus of his patient as being responsible for his eponymous aphasia (Broca, 1861). This contribution launched an emphasis on regions specialized for language, which were later conceptualized as a network consisting of Broca’s and Wernicke’s areas connected via the arcuate fasciculus (N. Geschwind, 1972).

Contemporarily, the language network is regarded as a prototypical example of a lateralized network, with left-hemisphere language lateralization estimated to occur in most (Breier et al., 2000;Stippich et al., 2003) to more than 90% of the general population (Corballis, 2003). The canonical language network is a distributed network comprising regions across the frontal, temporal, and parietal lobes, with lines of evidence stemming from a variety of sources including lesion cases (Broca, 1861;N. Geschwind, 1970;C. Wernicke, 1874), intraoperative brain stimulation (Penfield & Jasper, 1954), neurodegeneration (e.g., primary progressive aphasia;Amici et al., 2006;Gorno-Tempini et al., 2004;Mesulam et al., 2014,^2015^), task-based fMRI (Badzakova-Trajkov et al., 2010;Binder et al., 2011;Bishop et al., 2024;Fedorenko et al., 2010,^2011^;Friederici et al., 2000;Gerrits et al., 2020;Ivanova et al., 2021;Labache et al., 2020;Lipkin et al., 2022;Mazoyer et al., 2014;Scott et al., 2017;Skeide et al., 2016), functional transcranial Doppler (Bishop et al., 2024;Stroobant et al., 2009;Whitehouse & Bishop, 2009), and functional connectivity (Braga et al., 2020;Hacker et al., 2013;Lee et al., 2012). The typically asymmetric organization of this network in neurotypical individuals continues to be replicated (Elin et al., 2022;Malik-Moraleda et al., 2022;Olulade et al., 2020;Reynolds et al., 2019).

While the lateralization of language provides a compelling example, it also prompts broader questions about the origins and implications of cerebral lateralization across other cognitive domains. In attempting to unravel the origins of cerebral lateralization, researchers have explored theoretical perspectives ranging from the genetic and epigenetic (D. H. Geschwind & Miller, 2001;McManus, 1985) to interhemispheric conflict (Andrew et al., 1982;Corballis, 1991). One point of exploration has been the dynamic interactions of interdigitated lateralized and nonlateralized networks. Previously,Zago et al. (2016)examined the relationship between a highly right-lateralized visuospatial attention network and a highly left-lateralized language network within individuals. No correlation was identified between the laterality indices of these two networks in right handers and mixed handers, but a negative correlation was found within individuals with a strong left-hand preference. One potential mechanism for this finding stems from the corpus callosum. With connections between homotopic regions across the hemispheres (Chao et al., 2009), the corpus callosum may inhibit homologous activity which in turn facilitates the lateralization of the brain (Cook, 1984). It was suggested that in strong left handers, this potential mechanism may still be in play following early development in order to maintain a complementarity of functions between hemispheres (Zago et al., 2016).

An alternative explanation for lateralization can be found in interhemispheric conflict, in which competition for limited cortical resources during brain maturation may drive lateralization. According to this hypothesis, as different functional networks vie for cortical real estate and resources, they become lateralized. Alternatively, or in tandem with this mechanism, networks may become lateralized to optimize their efficiency, preventing interference from competing networks. Under this framework, as one network increases in lateralization to one hemisphere, that network occupies more space within that hemisphere while freeing up cortical territory in the contralateral hemisphere. Presumably, this would allow for a complementary network to become more lateralized in the contralateral hemisphere. One example of such a scenario may be found in a right-lateralized attention network composed of the temporoparietal junction and ventral frontal areas and which is hypothesized to process visuospatial information, particularly unexpected stimuli (Corbetta & Shulman, 2002). The ventral attention network in particular has been identified as a potential right-lateralized complement to the left-lateralized language network (Bernard et al., 2020).

Other functional networks in both the right and left hemispheres have been examined for evidence of lateralization. Of note, lateralization can be an indicator for specialization, or the dominant hosting of a macroscale functional network and its associated functional properties by one hemisphere over the other (Hervé et al., 2013). One study quantified specialization across seven functional networks and found that specialization was not restricted to a single left- or right-specialized network (D. Wang et al., 2014). Rather, the right frontoparietal network and right ventral and dorsal attention networks, as well as the left default and frontoparietal networks exhibited specialization as assessed via a functional connectivity-based metric (see Fig. 5;D. Wang et al., 2014). This pattern was generally replicated in highly sampled individuals, revealing left-lateralized language, default, and frontoparietal networks, as well as right-lateralized salience and frontoparietal networks (Braga et al., 2020). Interestingly, the finding of both left- and right-lateralized frontoparietal networks across bothBraga et al. (2020)andD. Wang et al. (2014)evidences a joint control system in which a subdivision of the frontoparietal control network is coupled with other lateralized networks in either the left or right hemisphere. Beyond this result, research on network lateralization has untapped potential when it comes to understanding the relationships between lateralized networks. This includes associations in laterality between ipsilateral and contralateral lateralized networks and extends to patterns within and across individuals.

In humans, hemispheric specialization has historically been identified using a variety of methods including callosotomy (i.e., split-brain patients; for review, seeGazzaniga, 2000), lateralized brain lesions (Milner, 1971;Rasmussen & Milner, 1977), the unilateral carotid administration of anesthetic (i.e., the Wada test;Wada & Rasmussen, 1960), and intraoperative brain stimulation mapping (Penfield & Jasper, 1954). Callosotomy studies have revealed the importance of interhemispheric communication for certain cognitive processes, demonstrating that the left and right hemispheres can operate relatively independently for some functions but require communication for others (Gazzaniga, 2000). Lateralized brain lesion studies, particularly the work of Milner and Rasmussen, have identified specific functions associated with each hemisphere, such as language processing predominantly in the left hemisphere (Milner, 1971;Rasmussen & Milner, 1977). Similarly, the Wada test has shed light on hemispheric dominance for language and memory (Wada & Rasmussen, 1960). Finally, leaning into the localization of specific functions to certain regions within each hemisphere, intraoperative brain stimulation mapping has provided detailed maps of functional areas in the brain (Penfield & Jasper, 1954). Collectively, these classic methods reveal patterns of human brain organization governed by interactions between lateralization and localization.

These historical methods are complemented by neuroimaging metrics, many of which are functional connectivity based. Of particular interest are the intrinsic laterality index (Liu et al., 2009), autonomy index (D. Wang et al., 2014), hemispheric contrast (Gotts et al., 2013), functional lateralization metric (Nielsen et al., 2013), classification metric (Friedrich et al., 2022), and network variants approach (Perez et al., 2023). Despite the unifying aim of estimating hemispheric specialization or lateralization, each of the listed methods varies in terms of how it approaches structural asymmetries, the addition of covariates such as handedness and gender, and short- and long-range connectivity.

In line with previous efforts to understand brain network organization and lateralization, the present study examines two questions. First, we explore which networks exhibit the greatest hemispheric asymmetries. A recent study involving 18 densely sampled individuals demonstrated that among 6 networks, the language network displayed the greatest left hemisphere lateralization, while a frontoparietal control network exhibited the greatest right hemisphere lateralization (Braga et al., 2020). However, it remains unclear how these estimates might change in a larger sample with a greater number of examined networks. Building upon the work ofBraga et al. (2020), we hypothesized that networks associated with language, visuospatial attention, and executive control would show the greatest hemispheric asymmetries.

Second, we investigate how lateralization in one network may influence the lateralization of other networks. We propose the following hypotheses to guide our investigation. The first hypothesis suggests that if an individual possesses a highly lateralized network, other networks for that individual will exhibit increased lateralization in the opposite direction, and that this dependent relationship will be systematic across individuals (the dependent hypothesis). The alternative hypothesis proposes that lateralization will be unrelated between networks across individuals (the independent hypothesis).

## 2 Methods

### 2.1 Datasets and overview

Three independent datasets were used for these analyses: The Human Connectome Project (HCP; split into discovery and replication datasets;Van Essen et al., 2013), the Human Connectome Project-Development (HCPD;Somerville et al., 2018), and the Natural Scenes Dataset (NSD;Allen et al., 2022). Each dataset was selected for its relatively high quantity of low-motion data per participant (seeFig. 1).

**Fig. 1.**
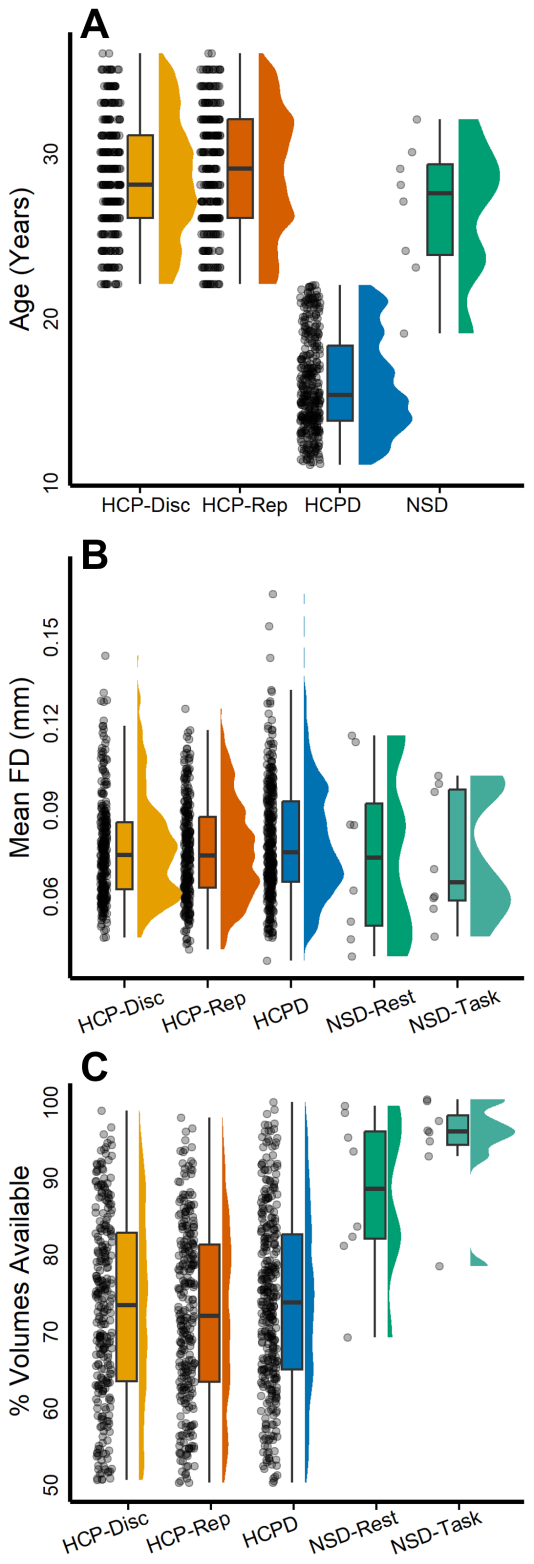
Participant age, data quality, and data availability. Panel (A) depicts participant age across each dataset following the implementation of exclusion criteria. HCP-Discovery participants included 276 individuals 22–36 years of age, HCP-Replication participants included 277 individuals 22–36 years of age, HCPD participants included 343 individuals 11–22 years of age, and NSD participants included 8 individuals 19–32 years of age. Panel (B) depicts the mean framewise displacement (FD) across each dataset following the implementation of exclusion criteria. HCP-Discovery mean FD was 0.08 mm (*SD*= 0.02 mm), range 0.04–0.14 mm; HCP-Replication mean FD was 0.07 mm (*SD*= 0.01 mm), range 0.04–0.12 mm; HCPD mean FD was 0.08 mm (*SD*= 0.02 mm), range 0.04–0.16 mm; NSD-Rest mean FD was 0.07 mm (*SD*= 0.03), range 0.04–0.11 mm; and NSD-Task mean FD was 0.07 mm (*SD*= 0.02 mm), range 0.04–0.1 mm. Panel (C) depicts the percentage of volumes remaining following motion-correction procedures for each dataset. HCP-Discovery mean percentage of volumes was 72.81% (*SD*= 12.12%), range 50.38–98.54%; HCP-Replication mean percentage of volumes was 72.09% (*SD*= 11.59%), range 50.04–97.62%; HCPD mean percentage of volumes was 73.6% (*SD*= 11.73%), range 50.05–99.63%; NSD-Rest mean percentage of volumes was 87.64% (*SD*= 10.51%), range 68.98–99.14%; and NSD-Task mean percentage of volumes was 94.27% (*SD*= 6.92), range 78.31–100%. Across each panel, a circle represents a single participant.

#### 2.1.1 HCP discovery and replication

The HCP S1200 release consists of 1,206 subjects (1,113 with structural MRI scans) collected at 13 different data acquisition sites with informed consent (Van Essen et al., 2013). Additional details regarding HCP scanning protocols are available online (https://humanconnectome.org/storage/app/media/documentation/s1200/HCP_S1200_Release_Appendix_I.pdf;Uğurbil et al., 2013;Van Essen et al., 2012). With a relatively large quantity of data available per individual, these data are ideally suited for taking a within-individual approach to specialization. Participants underwent four 15-min runs of a passive fixation task (resting-state fMRI) during which they were asked to keep their eyes open while viewing a white cross on a dark background and think of nothing in particular while remaining awake (Smith et al., 2013). Exclusion criteria for the HCP S1200 release included removing participants with a mean framewise displacement greater than 0.2 mm and mean DVARS greater than 50, participants missing handedness data, and participants with less than 50% of volumes remaining after motion censoring. This resulted in a subsample of 553 participants, which was divided into two equal parts, a discovery dataset and a replication dataset, using random sampling without replacement. The two datasets were then compared using the R package MatchIt (version 4.5.4;Ho et al., 2023) on age, mean framewise displacement, sex, handedness, and percentage of volumes remaining following motion censoring. The HCP-Discovery dataset consisted of 276 participants 22–36 years old (*M*= 28.48,*SD*= 3.58) with 167 females, while the HCP-Replication dataset consisted of 277 participants 22–36 years old (*M*= 28.7,*SD*= 3.77) with 173 females.

#### 2.1.2 HCPD

With a younger sample and smaller quantity of data per individual, the HCPD dataset was used as an additional replication dataset for primary analyses. Since data collection for the HCPD project is ongoing, cross-sectional data from the latest release were included, and these were composed of 652 healthy participants. All data were obtained with informed assent or consent. As a part of the HCPD protocol, participants underwent four 6.5-min runs of resting-state fMRI, with an exception for participants 5–7 years old, which had six 3.5-min runs each (Harms et al., 2018). Participants were instructed to view a small white fixation crosshair on a black background and blink normally. Exclusion criteria for HCPD included removing participants with less than 50% of volumes remaining after motion censoring, participants missing handedness data, and participants with a mean framewise displacement greater than 0.2 mm and mean DVARS greater than 50 (seeFig. 1). Following the exclusion criteria, the dataset consisted of 343 individuals ages 11–21.92 years (*M*= 15.93,*SD*= 2.97) of which 189 were female.

#### 2.1.3 NSD

With a large quantity of resting-state and task fMRI data available per individual, the NSD was included to examine potential task effects on estimating individual network parcellations and specialization. The NSD is composed of eight individuals (two males and six females; age range 19–32 years). All data were obtained with informed written consent according to the University of Minnesota institutional review board. As detailed inAllen et al. (2022), participants averaged 2 h of resting-state fMRI and 39.5 h of task-based fMRI. For the resting-state runs, participants were instructed to stay awake and fixate on a white cross placed on a gray background but otherwise rest. During the task-based runs, participants were shown distinct natural scenes taken from the Microsoft Common Objects in Context database (Lin et al., 2014). Images were presented for 3 s with 1-s gaps in between images. Subjects fixated centrally and performed a long-term continuous recognition task on the images. Exclusion criteria for NSD included removing participants with less than 50% of volumes remaining after motion censoring, and participants with a mean framewise displacement greater than 0.2 mm and mean DVARS greater than 50. No subjects were excluded from the analysis; however, following motion correction, a minimum of 12 resting-state fMRI runs (approximately 60 min) remained. To compare resting-state and task data on equal grounds, only the first 12 available resting-state runs and the first 12 available task fMRI runs from each participant were utilized.

### 2.2 MRI acquisition parameters

#### 2.2.1 HCP discovery and replication

The HCP dataset was acquired on a custom Siemens 3T Skyra with a 32-channel head coil. T1-weighted images were collected with a 3D MPRAGE sequence with isotropic 0.7 mm voxels (256 sagittal slices, repetition time [TR] = 2,400 ms, echo time [TE] = 2.14 ms) as detailed inGlasser et al. (2013). Resting-state functional images were collected using 2 mm isotropic voxels (72 sagittal slices, TR = 720 ms, TE = 33 ms, multiband accelerated pulse sequence with multiband factor = 8) as detailed inGlasser et al. (2013,^2016^).

#### 2.2.2 HCPD

The HCPD MRI data were acquired on Siemens 3T Prisma scanners with vendor 32-channel head coils at four sites: Harvard University, University of California-Los Angeles, University of Minnesota, and Washington University in St. Louis (Harms et al., 2018). Structural T1-weighted scans were acquired with a multiecho MPRAGE sequence (van der Kouwe et al., 2008) with 0.8 mm isotropic voxels (sagittal FOV = 256 × 240 × 166; matrix size = 320 × 300 × 208 slices; slice oversampling = 7.7%; 2-fold in-plane acceleration (GRAPPA); pixel bandwidth = 744 Hz/Px; Tr/TI = 2,500/1,000, TE = 1.9/3.6/5.4/7.2 ms, flip angle = 8°; water excitation employed for fat suppression; up to 30 TRs allowed for motion-induced reacquisition). T2*-weighted scans were used for resting-state fMRI with 2D multiband gradient-recalled echo echo-planar imaging sequence (MB8, TR/TE = 800/37 ms, flip angle = 52°) and 2.0 mm isotropic voxels covering the whole brain (72 oblique-axial slices). Functional scans were acquired in pairs of two runs with opposite phase encoding polarity (anterior-to-posterior and posterior-to-anterior) so that fMRI data were not biased toward either phase encoding polarity. For all scans, Framewise Integrated Real-time MRI Monitoring (Dosenbach et al., 2017) was implemented to provide motion feedback to participants between fMRI runs.

#### 2.2.3 NSD

The NSD dataset was acquired at the Center for Magnetic Resonance Research at the University of Minnesota (Allen et al., 2022). Anatomical data (such as T1-weighted volumes) were collected using a 3T Siemens Prisma scanner with a standard Siemens 32-channel RF head coil while functional data were collected using a 7T Siemens Magnetom passively shielded scanner and a single-channel-transmit, 32-channel-receive RF head coil. T1-weighted images were acquired with an MPRAGE sequence (0.8-mm bandwidth 220 Hz per pixel, no partial Fourier, in-plane acceleration factor (iPAT) 2, TA = 6.6 min per scan). Functional data were collected using gradient-echo EPI at 1.8-mm isotropic resolution with whole-brain coverage (84 axial slices, slice thickness 1.8 mm, slice gap 0 mm, field-of-view 216 mm (FE)×216 mm (PE), phase encode direction anterior-to-posterior, matrix size 120×120, TR = 1,600 ms, TE = 22.0 ms, flip angle 62°, echo spacing 0.66 ms, bandwidth 1,736 Hz per pixel, partial Fourier 7/8, iPAT 2, multiband slice acceleration factor 3). Full protocol printouts for the NSD dataset are available online (https://cvnlab.slite.page/p/NKalgWd__F/Experiments).

### 2.3 fMRI preprocessing

Preprocessing took place on raw NIFTI files for the resting-state fMRI and task fMRI runs using a pipeline developed by the Computational Brain Imaging Group (CBIG;R. Kong et al., 2019;Li et al., 2019; code is available online athttps://github.com/ThomasYeoLab/CBIG/tree/c773720ad340dcb1d566b0b8de68b6acdf2ca505/stable_projects/preprocessing/CBIG_fMRI_Preproc2016). This CBIG2016 preprocessing pipeline was selected to process the fMRI data in order to more closely follow the processing steps used to implement the multisession hierarchical Bayesian modeling parcellation method (R. Kong et al., 2019). As a prerequisite, this pipeline requires FreeSurfer recon-all output from the structural data (FreeSurfer 6.0.1, RRID:SCR_001847;Dale et al., 1999). The fMRI data are then processed with the following steps: (1) removal of the first four frames and (2) motion correction using rigid body translation and rotation with the FSL package (RRID:SCR_002823;Jenkinson et al., 2002;Smith et al., 2004). The structural and functional images are then aligned using boundary-based registration (Greve & Fischl, 2009) using the FsFast software package (http://surfer.nmr.mgh.harvard.edu/fswiki/FsFast). FD and DVARS were computed using*fsl_motion_outliers*(Smith et al., 2004). Volumes with FD > 0.2 mm or DVARS > 50 were tagged as outliers. Uncensored segments of data lasting fewer than five contiguous volumes were also flagged as outliers (Gordon et al., 2016). BOLD runs with more than half of the volumes flagged as outliers were removed completely. Next, linear regression using multiple nuisance regressors was applied through a combination of CBIG in-house scripts and the FSL MCFLIRT tool (Jenkinson et al., 2002). Nuisance regressors consisted of global signal, 6 motion correction parameters, averaged ventricular signal, averaged white matter signal, and their temporal derivatives (totaling 18 regressors). The flagged outlier volumes were ignored during the regression procedure. Following the regression, a bandpass filter (0.009 Hz ≤ f ≤ 0.08 Hz) was applied using CBIG in-house scripts. At this point, the preprocessed fMRI data were projected onto the FreeSurfer fsaverage6 surface space (2 mm vertex spacing) with FreeSurfer’s*mri_vol2surf*function. The projected fMRI data were then smoothed using a 6 mm full-width half-maximum kernel through FreeSurfer’s*mri_surf2surf*function (Fischl et al., 1999). Surface space was selected for the following analyses in order to best follow the individual parcellation pipeline outlined inR. Kong et al. (2019), and following evidence that landmark surface-based registration outperforms volume-based registration (Anticevic et al., 2008;Argall et al., 2006;Desai et al., 2005;Van Essen, 2005).

### 2.4 Individual network parcellation

Following preprocessing, network parcellations were computed using a multisession hierarchical Bayesian modeling (MS-HBM) pipeline. The MS-HBM pipeline is designed to generate parcellations for individuals with multiple sessions of fMRI data (R. Kong et al., 2019) and this 2019 version of the pipeline was implemented in MATLAB R2018b (RRID:SCR_001622;MATLAB, 2018). This particular model was selected because it accounts for intraindividual variation, allowing the model to better generalize to new fMRI data from the same participant. As an overview, this model uses a variational Bayes expectation-maximization algorithm to learn group-level priors from a training dataset and then apply those to estimate individual-specific parcellations (seeFig. 2). This model estimates the following parameters: group-level network connectivity profiles, intersubject functional connectivity variability, intrasubject functional connectivity variability, a spatial smoothness prior, and an intersubject spatial variability prior. As recommended in the pipeline’s GitHub documentation, subjects with a single available run postpreprocessing had that single run split in two and a connectivity profile was generated for each split. A*k*of 17 was selected for all participants (Yeo et al., 2011). Additionally, it has previously been demonstrated that MS-HBM parameters estimated from one dataset can be effectively applied to another dataset with significant differences in acquisition and preprocessing (R. Kong et al., 2021). Thus, to generate our model, priors trained on 37 Genomic Superstruct Project (GSP) subjects were utilized (Holmes et al., 2015;R. Kong et al., 2019). Following the generation of individual parcellations, a Hungarian matching algorithm was used to match the clusters with theYeo et al. (2011)17-network group parcellation.

**Fig. 2.**
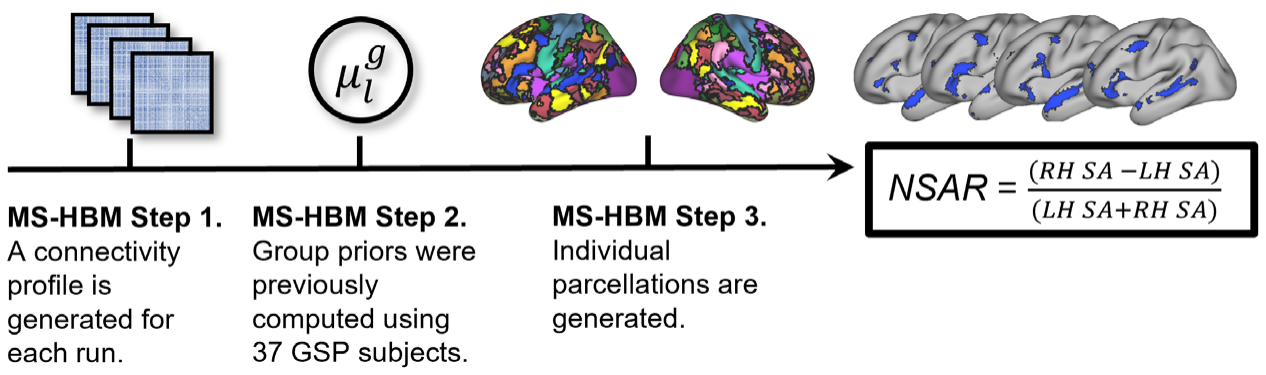
Illustration of the Multi-Session Hierarchical Bayesian Modeling (MS-HBM) individual parcellation pipeline. First, a connectivity profile is generated for each available fMRI run on an individual basis (illustrated here as a functional connectivity matrix). Next, group priors previously estimated (R. Kong et al., 2019) from 37 Genomic Superstruct Project (GSP) subjects were used. Third, the connectivity profiles from each available run and the group priors (more specifically, the intersubject functional connectivity variability, intrasubject functional connectivity variability, spatial smoothness, and intersubject spatial variability) are used to generate network parcellations for each participant. Finally, the network surface area ratio (NSAR) is calculated using the formula shown, where LH SA is the left hemisphere surface area for a given network and RH SA is the right hemisphere surface area for a given network. A negative NSAR value indicates left hemisphere lateralization for a given network while a positive value indicates right hemisphere lateralization.

### 2.5 Network surface area ratio

Following the generation of individual network parcellations, lateralization was estimated using the network surface area ratio (NSAR). In discussing this measure, we opted to use this terminology (lateralization) because it encapsulates the concept of an asymmetrical distribution of functional networks across the cerebral hemispheres, which is central to the following analyses. This measure was calculated within each individual for each of 17 networks by first extracting each network label as a region of interest using the Connectome Workbench wb_command functions*metric-label-import*and*gifti-label-to-roi*(Marcus et al., 2013). Next, the left and right hemisphere surface areas for a given network were calculated on a midthickness Conte69 surface in fsaverage6 resolution (Glasser & Essen, 2011) using the wb_command function*metric-stats*. Finally, NSAR was calculated as the difference between normalized left and right hemisphere surface areas for a given network (seeFig. 2):

$$\text{NSAR }=\frac{RHSA-LHSA}{LHSA+RHSA},$$

where RH SA represents the right hemisphere surface area of a given network and LH SA represents the left hemisphere surface area of a given network. A scaling factor was not included in the denominator since asymmetry indices including a scaling factor deliver essentially the same findings as those without (X.-Z. Kong et al., 2022).

NSAR values range from -1.0 to +1.0, with negative values indicating left hemisphere lateralization for a given network and positive values indicating right hemisphere lateralization. NSAR values closer to zero indicate less lateralization (i.e., hemispheric symmetry). NSAR is a threshold-independent measure of lateralization (i.e., not reliant upon determining a threshold*p*-value at which to view and analyze task-activation maps), the benefits of which have been previously discussed (Bradshaw et al., 2017). Although this measure of lateralization shares similarities with several previously used asymmetry indices (Binder et al., 1997;Braga et al., 2020;Mahowald & Fedorenko, 2016), its distinct methodology prompted us to specifically test its validity and reliability. The results of these reliability and validity analyses are presented in the following two sections (see theSupplementary Methodssection for details regarding analysis design).

### 2.6 NSAR as a valid measure of lateralization

The ecological validity of NSAR was examined through comparison against laterality calculated from a language comprehension task in a subset of the HCP subjects (*N*= 221). A positive significant relationship between NSAR for the language network and language comprehension task laterality was found (Spearman rank correlation ρ = 0.24,*p*< .001; seeSupplementary Fig. S1).

The convergent validity of NSAR was assessed through comparison with an additional functional measure of specialization (the autonomy index) using the Spearman rank correlation. To facilitate direct comparison with NSAR values, the sign for autonomy index values was reversed. With the selected left-lateralized networks, significant relationships were found between the autonomy index and NSAR for the Language (Spearman rank correlation ρ = -0.62,*p*< .001; seeFig. S1B), Dorsal Attention-A (Spearman rank correlation ρ = -0.62,*p*< .001), and the Control-B (Spearman rank correlation ρ = -0.57,*p*< .001) networks (see the top row ofSupplementary Fig. S2). Significant relationships were also found between the autonomy index and NSAR for the selected right-lateralized networks including the Visual-B (Spearman rank correlation ρ = 0.71,*p*< .001), Salience/Ventral Attention-A (Spearman rank correlation ρ = 0.61,*p*< .001), and Limbic-B (Spearman rank correlation ρ = 0.69,*p*< .001) networks (see the second row ofSupplementary Fig. S2). These findings indicate that NSAR and the autonomy index are measuring similar facets of specialization.

Next, the external validity of NSAR was examined through comparison against two cognitive measures using a Canonical Correlation Analysis (CCA): a reading task (ORRT) and an attention/inhibitory control task (the Flanker task). In preparation for the CCA in a subset of HCP participants (*N*= 232; no missing data), linearity and heteroskedasticity of age-, sex-, handedness-, and mean framewise displacement-adjusted NSAR values from eight networks (Visual-B, Language, Dorsal Attention-A, Salience/Ventral Attention-A, Control-B, Control-C, Default-C, and Limbic-B) and the age- and sex-adjusted values from two cognitive measures were evaluated in pairwise plots, which were followed by the Doornik–Hansen multivariate test for normality (*DH*= 164.21,*p*= 0). Tests of dimensionality for the CCA indicated that one of the two canonical dimensions was statistically significant at the .05 level. This dimension had a canonical correlation of 0.34 (*F*(16, 444) = 0.87,*p*= .008) between the cognitive measures and NSAR values, while the canonical correlation was much lower for the second, nonsignificant dimension at 0.14 (*F*(7, 223) = 0.98,*p*= .75).Supplementary Table S1presents the standardized canonical coefficients for the first dimension across the cognitive measures and eight networks. Of the cognitive variables, the first canonical dimension was most strongly influenced by language ability (β (standardized canonical coefficient) = -0.99). In terms of networks, the Visual-B (β = -0.33,*r*= -0.13), Language (β = 0.39,*r*= 0.2), Dorsal Attention-A (β = -0.54,*r*= -0.17), and Control-C (β = 0.48,*r*= 0.13) networks appeared to contribute the most to the first canonical dimension. Haufe-transformed feature weights indicated that for every one-unit increase in language network lateralization, the first dimension, representing language ability, increases by 0.13 (seeSupplementary Table S1). These findings suggest that there is a relationship between network lateralization and cognitive abilities, specifically language.

### 2.7 NSAR as a reliable measure of lateralization

#### 2.7.1 Stable estimate analysis

To address the question of how much data are needed to obtain a stable estimate of NSAR values, combinations of 5-min increments (5, 10, 15…30 min) were compared against 30 independent minutes of data in a subset of HCP subjects. The intraclass correlations indicate that only 5 min of data are needed to obtain moderate-to-good intraclass correlations for the majority of subjects (seeSupplementary Fig. S3A). Of note, poor and excellent interclass correlations were observed for some subjects. The stable estimate analysis was also approached from a network basis (as opposed to the subject basis presented inSupplementary Fig. S3A). Networks with the lowest intraclass correlations included the Limbic-A and Control-A networks, while networks with the greatest intraclass correlations included Visual-A, Limbic-B, and Default-A (for overall distributions, seeSupplementary Fig. S3B; for specific network intraclass correlation coefficients, seeSupplementary Fig. S4). Interestingly, not all networks improved in reliability with additional data, including the Limbic-A and Control-A networks. This is likely a reflection of a poor signal-to-noise ratio.

Next, parcellation label overlap estimates were computed. When a single dice coefficient was calculated within individuals, only 5 min of data were needed to obtain a mean dice coefficient of 0.75 (*SD*= 0.03), which was highly similar to the 0.76 mean (*SD*= 0.03) dice coefficient obtained with 30 min of data (seeSupplementary Fig. S5A). Networks with the highest mean dice coefficients include those related to sensory and somatomotor functions such as the Visual-A, Visual-B, Somatomotor-A, and Somatomotor-B networks (seeSupplementary Fig. S5B).

#### 2.7.2 Test–retest reliability analysis

Using HCP subjects with all four resting-state runs available postpreprocessing (*N*= 232), test–retest reliability was assessed for three left-lateralized networks (Language, Dorsal Attention-A, and Control-B) and three right-lateralized networks (Limbic-B, Visual-B, and Salience/Ventral Attention-A) determined*a priori*. For the left-lateralized networks, intraclass correlations were within the moderate range, from 0.56 to 0.63, with the lowest being the Dorsal Attention-A network (ICC = 0.56,*F*(231, 231) = 3.6,*p*< .001, 95% CI [0.47, 0.64]; seeSupplementary Fig. S6). For the right-lateralized networks, intraclass correlations remained in the moderate range, between 0.58 and 0.71, with the Visual-B network exhibiting the lowest reliability (ICC = 0.58,*F*(231, 231) = 3.7,*p*< .001, 95% CI [0.48, 0.66]).

#### 2.7.3 Task effects on individual parcellations and NSAR

Using the NSD dataset (*N*= 8) to compare potential differences between resting-state and task fMRI on individual parcellations and NSAR estimates, we found differences between the within-task comparisons and between-task comparisons for both the parcellation dice coefficients and NSAR intraclass correlations (seeSupplementary Fig. S7). Wilcoxon signed rank comparisons revealed a difference in within-task (Task-Task and Rest-Rest) dice coefficients for even versus odd numbered runs (*V*= 36,*p*= .008), but no difference for the first half versus the second half of runs (*V*= 29,*p*= .15) or the random selection of runs (*V*= 31,*p*= .08). Regardless of how the data were split, a task effect in dice coefficient was found between within-task (Task-Task) and between-task (Task-Rest) dice coefficients for even versus odd numbered runs (*V*= 36,*p*= .008), the first half versus the second half of runs (*V*= 36,*p*= .008), and the random selection of runs (*V*= 36,*p*= .008).

Similarly, with the NSAR intraclass coefficients, no significant difference was found for within-task (Task–Task and Rest–Rest) reliability across the even versus odd numbered runs (*V*= 31,*p*= .08) and the first half versus the second half of runs (*V*= 19,*p*= .95), but not for the random selection of runs (*V*= 35,*p*= .02). However, no significant difference was found between within-task (Task–Task) and between-task (Task–Rest) intraclass correlation coefficients across the even versus odd numbered runs (*V*= 31,*p*= .08), the first half versus the second half of runs (*V*= 31,*p*= .08), but for the random selection of runs (*V*= 34,*p*= .02).

### 2.8 Identifying lateralized networks

After examining validity and reliability, we addressed the first hypothesis of determining whether any of the 17 networks exhibited lateralization, and of those, which were the most lateralized. The following analyses were first implemented in the HCP-Discovery dataset and then replicated in the HCP-Replication and HCPD datasets using all data available from each participant. First, to determine whether any networks exhibited lateralization, multiple regressions were implemented for each of the 17 networks. Models consisted of a given network’s NSAR value and the covariates of mean-centered age, sex, mean-centered mean framewise displacement, and handedness (measured via the Edinburgh Handedness Inventory;Oldfield, 1971). A network was considered lateralized if the model intercept was significant at the Bonferroni-corrected alpha level of 0.003 (derived from dividing 0.05 by 17 networks). Next, to determine which networks were the most lateralized, any networks exhibiting significant lateralization in the previous tests with the same direction of lateralization were compared against each other two at a time in multiple regressions with a binary variable for the two networks and the covariates of mean-centered age, sex, mean-centered mean framewise displacement, and handedness.

### 2.9 Identifying network relationships

To test the second hypothesis regarding how network lateralization is potentially related between networks, a general relationship was first assessed between NSAR values averaged across like-lateralized networks followed by correlation matrices and structural equation modeling. An exploratory factor analysis (EFA) was conducted in the HCP-Discovery dataset followed by separate confirmatory factor analyses (CFAs) in the HCP-Replication and HCPD datasets using model-adjusted lateralization values from any reliably lateralized networks. For a network to be considered reliably lateralized, it was significantly lateralized across the HCP-Discovery, HCP-Replication, and HCPD datasets. An EFA is a statistical technique used to identify underlying factors or dimensions within a set of variables without preconceived hypotheses. Thus, the EFA was chosen for its ability to identify shared relationships between items in a data-driven manner. Similarly, a CFA is used to test a specific theory or model by assessing how well the observed data fit the hypothesized factor structure. Accordingly, the CFA analyses performed in the HCP-Replication and HCPD datasets were used to test an explicit hypothesis based on the results from the EFA in the HCP-Discovery dataset.

In preparation for the EFA, the linearity and heteroskedasticity of adjusted NSAR values from the lateralized networks were evaluated in pairwise plots, which were followed by the Doornik–Hansen multivariate test for normality (*DH.test*function from the mvnTest package; version 1.1.0;Doornik & Hansen, 2008;Pya et al., 2016). If the Doornik–Hansen test yields a small*p*-value, we reject the null hypothesis of multivariate normality; a large*p*-value suggests that we do not reject the null hypothesis, indicating consistency with multivariate normality. NSAR values were then evaluated for multicollinearity using the Variance Inflation Factor (*vif*function from the psych package; version 2.2.5;Revelle, 2023). A low Variance Inflation Factor value (in general, scores below 4 or even 10 are considered acceptable) indicates that the examined variables are not highly correlated, meeting the assumption of independence crucial for factor analysis. Additional assumptions testing next included Bartlett’s test of sphericity and the Kaiser–Meyer–Olkin (KMO) Measure of Sampling Adequacy. Bartlett’s test of sphericity assesses whether the correlation matrix of variables is significantly different from an identity matrix. A significant result indicates that the null hypothesis of orthogonality is rejected, indicating that variables are correlated and thus suitable for factor analysis. The KMO test assesses the sampling adequacy for factor analysis by examining the correlations between variables. A KMO value closer to 1 indicates better suitability.

Next, the*fa*function from the psych package (Revelle, 2023) was used to conduct an iterated principal factors analysis and subsequent parallel analysis. Criteria for the extraction of factors were a minimum eigenvalue of 1, visual inspection of a scree plot, and a parallel analysis. A four-factor model was hypothesized, similar toLiu et al. (2009), with each factor encompassing vision, internal thought, attention, and language. The factor structure identified in the HCP-Discovery dataset was then implemented in confirmatory factor analyses in the HCP-Replication and HCPD datasets using the*cfa*function from the lavaan package (version 0.6.15;Rosseel, 2012;Rosseel et al., 2023).

## 3 Results

### 3.1 Networks with the greatest lateralization

To test the first hypothesis that networks associated with language, visuospatial attention, and executive control would show the greatest hemispheric lateralization, networks were first evaluated for lateralization and then compared against each other. To begin, a series of multiple regressions were used to identify whether any of the 17 networks were lateralized, first in the HCP-Discovery dataset and then in the HCP-Replication and HCPD datasets. Nine networks showed significant lateralization (*p*< .003) consistently in the same direction (e.g., right or left lateralization) across all three datasets. Among these networks, four were left lateralized (Language, Dorsal Attention-A, Control-A, and Default-C), and five were right lateralized (Visual-B, Salience/Ventral Attention-A, Control-B, Control-C, and Limbic-B; refer toSupplementary Table S2). However, given the very low reliability of the left-lateralized Control-A network (mean ICC = 0.12; seeSupplementary Fig. S4), this network was not considered further (overlap maps for the remaining eight lateralized networks across the three datasets are depicted in Supplementary Figs. S8–S10). None of the covariates were reliably significant for a given network across all three datasets (seeSupplementary Fig. S11for a depiction of the lack of age effects in the HCPD dataset specifically). SeeFigure 3for model-adjusted NSAR values for each of the 17 networks.

**Fig. 3.**
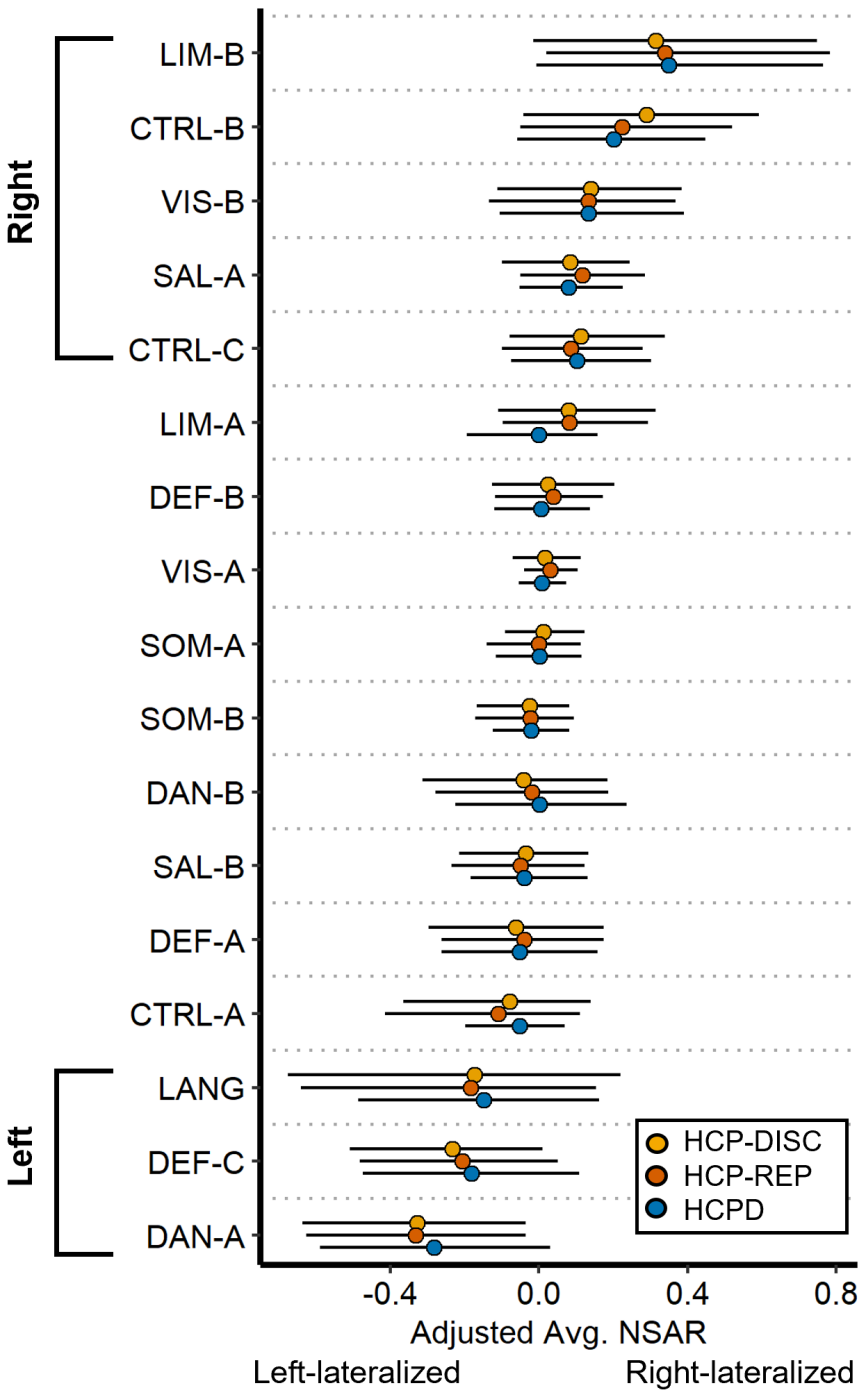
Lateralization for 17 networks across the HCP-Discovery, HCP-Replication, and HCPD datasets. On the y-axis are the 17 networks and on the x-axis are the adjusted NSAR values, with negative values representing left hemisphere lateralization and positive values representing right hemisphere lateralization. Bars represent the 2.5 and 97.5 percentiles. NSAR values were adjusted by regressing out the effects of mean-centered age, mean-centered mean framewise displacement, and sex using the following formula: NSAR_adjusted_= NSAR_raw_– [β_1_(mean-centered age_raw_– mean of mean-centered age_raw_) + β_2_(mean-centered FD_raw_– mean of mean-centered FD_raw_) + β_3_(sex_raw_– mean sex_raw_) + β_4_(handedness_raw_– mean handedness_raw_)]. NSAR adjustment occurred separately for each network within each dataset. Lines represent the standard error. Across the three datasets, eight networks were reliably and significantly lateralized (left lateralized: Language, Dorsal Attention-A, and Default-C; right lateralized: Visual-B, Salience/Ventral Attention-A, Control-B, Control-C, and Limbic-B).

Following the identification of eight lateralized networks, a series of multiple regressions was used to compare networks with the same direction of lateralization two at a time in order to identify the networks with the greatest lateralization. Models included a binary network variable and the covariates of mean-centered age, sex, handedness, and mean-centered mean framewise displacement. Of the left-lateralized networks, the Dorsal Attention-A network was the most lateralized compared with the Language and Default-C networks, and this pattern was replicated across the HCP-Discovery, HCP-Replication, and HCPD datasets (seeSupplementary Table S3). Of the right-lateralized networks, the Limbic-B network was the most lateralized, followed by the Control-B network, Visual-B and Control-C networks (not significantly different), and the Salience/Ventral Attention-A network. This pattern was replicated across the three datasets as well (seeSupplementary Table S4). Contrary to our hypothesis that networks associated with language, visuospatial attention, and executive control would show the greatest lateralization, we identified the Dorsal Attention-A network as the most left lateralized and the Limbic-B network as the most right lateralized.

### 3.2 Relationships between networks’ lateralization

Next, we investigated how lateralization in one network may influence the lateralization of other networks. This second hypothesis was initially evaluated using general correlations, and then further examined using correlation matrices and structural equation modeling. These assessments were performed across the HCP-Discovery, HCP-Replication, and HCPD datasets. First, model-adjusted NSAR values were averaged across like-lateralized networks before the averaged left-lateralized values (from the Language, Dorsal Attention-A, and Default-C networks) were correlated with the averaged right-lateralized values (from the Visual-B, Salience-Ventral Attention-A, Control-B, Control-C, and Limbic-B networks). A general negative relationship between left-lateralized and right-lateralized networks was found across each dataset (HCP-Discovery:*r*(274) = -0.67,*p*< .001; HCP-Replication:*r*(275) = -0.59,*p*< .001; HCPD:*r*(343) = -0.66,*p*< .001). Next, correlation matrices of the model-adjusted NSAR values from the eight lateralized networks evidenced moderate negative relationships between the left- and right-lateralized networks across individuals (seeFig. 4). In the HCP-Discovery dataset, negative relationships were found between the Limbic-B and Dorsal Attention-A networks (*r*(274) = -0.45,*p*< .001, 95% CI [-0.54, -0.36]; seeFig. 5A), the Limbic-B and Default-C networks (*r*(274) = -0.42,*p*< .001, 95% CI [-0.51, -0.31]; seeFig. 5B), the Default-C and Visual-B networks (*r*(274) = -0.16,*p*= .007, 95% CI [-0.28, -0.05]), the Default-C and Control-B networks (*r*(274) = -0.27,*p*< . 001, 95% CI [-0.38, -0.16]), the Default-C and Control-C networks (*r*(274) = -0.17,*p*= .004, 95% CI [-0.29, -0.06]), the Control-B and Language networks (*r*(274) = -0.31,*p*< .001, 95% CI [-0.41, -0.19]), and the Language and Salience/Ventral Attention-A networks (*r*(274) = -0.3,*p*< .001, 95% CI [-0.41, -0.19]; seeFig. 5C). Interestingly, a negative relationship was also found between two left-lateralized networks: Dorsal Attention-A and Language (*r*(274) = -0.23,*p*< .001, 95% CI [-0.34, -0.11]; seeSupplementary Fig. S12for the functional neuroanatomy of these networks). Each negative relationship was replicated across the HCP-Replication and HCPD datasets (seeFig. 4). These relationships support the dependent hypothesis, which suggests that having one highly lateralized network corresponds with increased lateralization in other networks within the individual, and that this pattern is systematic across individuals.

**Fig. 4.**
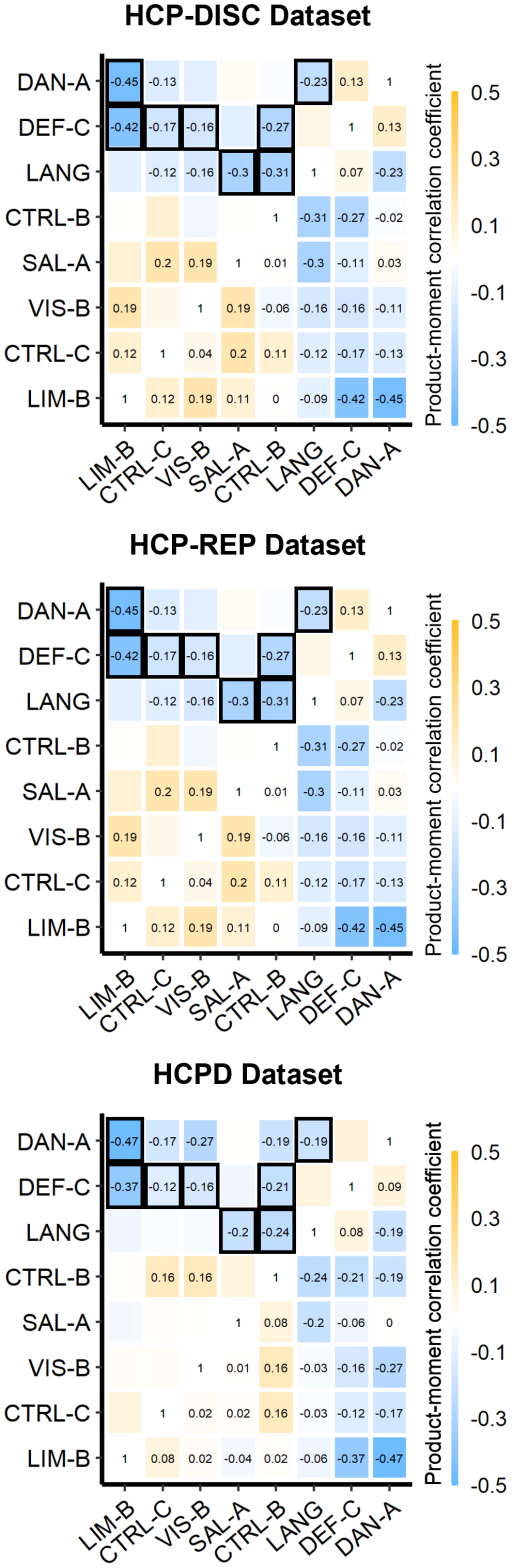
Relationships between lateralized networks across the HCP-Discovery, HCP-Replication, and HCPD datasets. Correlation matrices were created from the model-adjusted NSAR values from the eight lateralized networks (Visual-B, Language, Dorsal Attention-A, Salience/Ventral Attention-A, Control-B, Control-C, Default-C, and Limbic-B), controlling for sex, mean-centered age, mean-centered framewise displacement, and handedness. Correlation values thresholded at*p*= .05 are displayed in the upper triangles, and consistent relationships have been highlighted with black boxes.

**Fig. 5.**
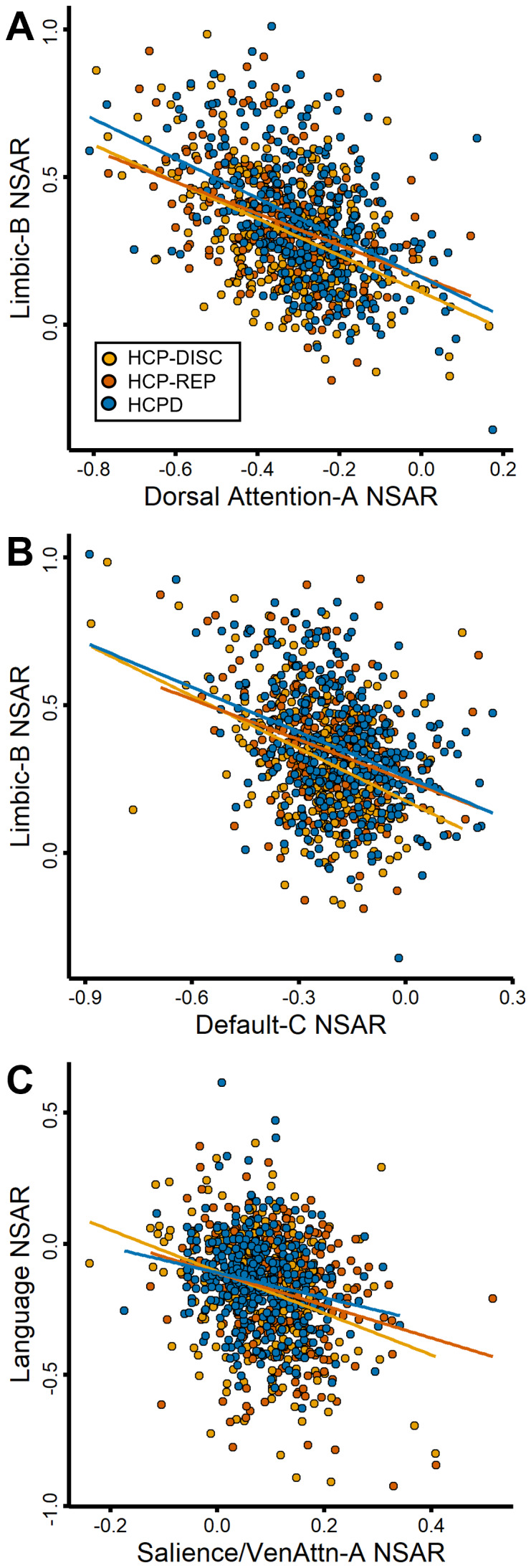
Negative correlations between highly left- and right-lateralized networks across the HCP-Discovery, HCP-Replication, and HCPD datasets. Panel (A) depicts the negative relationship between the Limbic-B and Dorsal Attention-A networks (HCP-Discovery:*r*(274) = -0.45, adjusted*R^2^*= 0.2; HCP-Replication:*r*(275) = -0.41, adjusted*R^2^*= 0.16; HCPD:*r*(341) = -0.47, adjusted*R^2^*= 0.22). Panel (B) depicts the negative relationship between the right-lateralized Limbic-B and left-lateralized Default-C networks (HCP-Discovery:*r*(274) = -0.42, adjusted*R^2^*= 0.17; HCP-Replication:*r*(275) = -0.31, adjusted*R^2^*= 0.09; HCPD:*r*(341) = -0.37, adjusted*R^2^*= 0.14). Panel (C) depicts the negative relationship between the right-lateralized Salience/Ventral Attention-A network and left-lateralized Language network (HCP-Discovery:*r*(274) = -0.3, adjusted*R^2^*= 0.09; HCP-Replication:*r*(275) = -0.25, adjusted*R^2^*= 0.06; HCPD:*r*(341) = -0.2, adjusted*R^2^*= 0.04). In each panel, a circle represents a single participant’s model-adjusted NSAR value, which was adjusted for mean-centered age, sex, handedness, and mean-centered mean framewise displacement.

#### 3.2.1 EFA in the HCP-Discovery dataset

As an additional method for exploring relationships between lateralized networks, an EFA was implemented in the HCP-Discovery dataset, followed by CFAs in the HCP-Replication and HCPD datasets. In preparation for the EFA in the HCP-Discovery dataset (*N*= 276), linearity and heteroskedasticity of adjusted NSAR values from the eight lateralized networks were evaluated in pairwise plots, which were followed by the Doornik–Hansen multivariate test for normality (*DH*= 202.89,*p*= 0). The NSAR values were then evaluated for multicollinearity, and no items had Variance Inflation Factor values greater than 1.65. Next, for the test of sphericity, we rejected the null hypothesis that there is no correlation among the items (χ^2^(28) = 293.43,*p*< .001). Additionally, the KMO test was 0.46, revealing that the extracted factors will account for an unacceptable amount of common variance.

To examine network relationships, a principal factors analysis in the HCP-Discovery dataset was performed. Using the correlation matrix from eight lateralized networks, two factors were extracted. This first factor had an eigenvalue of 1.38 (explaining 57% of the variance; seeTable 1for factor loadings) and the second factor had an eigenvalue of 1.02 (explaining 43% of the variance). Of note, the left-lateralized networks load negatively onto the first extracted factor, while right-lateralized networks load positively, suggesting that this factor encompasses right-hemisphere lateralization, with the opposite in the second extracted factor.

**Table 1.** Summary of exploratory factor analysis results for the NSAR scores using iterated principal factors in the HCP-Discovery dataset (N = 276).

Network	Factor 1 Loadings	Factor 2 Loadings
Limbic-B	**0.73**	-0.32
Control-C	0.28	0.06
Visual-B	0.29	0.04
Salience/VenAttn-A	0.28	0.26
Control-B	0.22	0.23
Language	-0.38	**-0.75**
Default-C	**-0.51**	0.05
Dorsal Attention-A	-0.37	**0.48**
Eigenvalues	1.38	1.02
Proportion of variance explained	0.57	0.43

*Note:*Factor loadings over 0.40 appear in bold.

#### 3.2.2 CFAs in the HCP-Replication and HCPD datasets

In preparation for the CFA in the HCP-Replication dataset (*N*= 277), linearity and heteroskedasticity of adjusted NSAR values were evaluated in pairwise plots, which were followed by the Doornik–Hansen multivariate test for normality (*DH*= 43.29,*p*< .001). The NSAR values were then evaluated for multicollinearity, and no items had Variance Inflation Factor values greater than 1.3. Next, for the test of sphericity, we rejected the null hypothesis that there is no correlation among the items (χ^2^(6) = 101.53,*p*< .001). Additionally, the KMO test was 0.49, revealing that the extracted factors will account for an unacceptable amount of common variance. This process of evaluating assumptions was also performed in the HCPD dataset (*N*= 343), starting with pairwise plots and the Doornik–Hansen multivariate test for normality (*DH*= 44.37,*p*< .001). Multicollinearity was then evaluated, and no items had Variance Inflation Factor values greater than 1.49. Additionally, for Bartlett’s test of sphericity, we rejected the null hypothesis that there is no correlation among the items (χ^2^(6) = 164.59,*p*< .001). Furthermore, the KMO test was 0.47, revealing that the extracted factors will account for an unacceptable amount of common variance.

To examine the relationships between lateralized networks and potentially replicate the HCP-Discovery EFA, a confirmatory factor analyses were performed in the HCP-Replication and HCPD datasets. The structural model consisted of two factors, with Limbic-B and Default-C loaded onto the first factor and Language and Dorsal Attention-A loaded onto the second factor. In the HCP-Replication dataset, the model provided fair fit to the data: χ^2^(2) = 61.95,*p*< .001; confirmatory fit index (CFI) = 0.38; root-mean-square error of approximation (RMSEA) = 0.33; standardized root mean square residual (SRMR) = 0.14. Similar results were found in the HCPD dataset, for which the model provided fair fit to the data: χ^2^(2) = 102.02,*p*< .001; CFI = 0.38; RMSEA = 0.38; SRMR = 0.16. Standardized loadings for each network across both CFAs are shown inTable 2.

**Table 2.** Standardized loadings for a two-factor confirmatory factor analysis model of NSAR scores in the HCP-Replication (N = 277) and HCPD (N = 343) datasets.

	HCP-Replication		HCPD	
Network	Factor 1	Factor 2	Factor 1	Factor 2
Limbic-B	-0.3	0	-0.36	0.00
Language	0.00	**0.84**	0.00	**0.91**
Default-C	**1.03**	0.00	**1.03**	0.00
Dorsal Attention-A	0.00	-0.25	0.00	-0.21

*Note:*Factor loadings over 0.40 appear in bold.

## 4 Discussion

In this study, we implemented a novel measure of lateralization based on the surface areas within high-resolution individual network parcellations (NSAR). Using NSAR, we identified eight networks that were reliably lateralized across three independent datasets. Furthermore, we examined potential relationships between networks’ NSAR values and found evidence supporting a dependent hypothesis of lateralization. These findings shed new light on hemispheric specialization, which has implications for the understanding of brain organization and development (Toga & Thompson, 2003), individual differences (Perez et al., 2023), human-defining cognitive processes (Hartwigsen et al., 2021), and neurodevelopmental conditions (Eyler et al., 2012;X.-Z. Kong et al., 2022).

### 4.1 The nature of NSAR and anatomical asymmetries

In this study, we examined lateralization using a novel surface area-based index that bridges both functional and structural aspects of the brain. While functional data were used to derive each network’s boundaries, NSAR is ultimately the ratio of a network’s left and right hemisphere surface areas, and surface area is a fundamentally structural measurement. Despite the identification of anatomical asymmetries in the brain for over a century (beginning with lesion work byBroca, 1861;Dax, 1865;C. Wernicke, 1874), it is still an open question as to which structures are on average asymmetrical in healthy individuals (X.-Z. Kong et al., 2022). Regardless, several major structural asymmetries have been reliably identified (for reviews, seeDuboc et al., 2015;X.-Z. Kong et al., 2022;Kuo & Massoud, 2022;Toga & Thompson, 2003). One such asymmetry includes Yakovlevian torque, which is a rotational asymmetry that describes how the right hemisphere juts forward anteriorly beyond the left, and the left hemisphere extends posteriorly beyond the right (LeMay, 1976). It has been proposed that this torque may be related to a fronto-occipital gradient of cortical thickness observed in 17,141 individuals from 99 datasets (X.-Z. Kong et al., 2018). This asymmetrical gradient includes greater cortical thickness in the left hemisphere for the frontal, primary sensory, superior parietal, and medial temporal cortices, and greater rightward thickness asymmetry observed in the posterior cortex (X.-Z. Kong et al., 2018). Another prominent structural asymmetry has been identified with the Sylvian fissure, which has been found to be significantly longer in the left hemisphere than in the right, although the right Sylvian fissure curves upward more anteriorly than the left and the left has a gentler slope (N. Geschwind & Levitsky, 1968). Inferior to the Sylvian fissure, an asymmetry has been identified with the superior temporal sulcus, which extends further into the brain in the right hemisphere than in the left hemisphere (Van Essen, 2005). This asymmetry is present in humans regardless of sex, age, or handedness (Leroy et al., 2015). Similarly,N. Geschwind and Levitsky (1968)also identified an asymmetry in the planum temporale, for which 65% of adults had greater gray matter volume in the left hemisphere than in the right. It was later found that language dominance (determined via the Wada test) was correlated with planum temporale asymmetry (Foundas et al., 1995). Heschl’s gyrus also exhibits a leftward asymmetry but for surface area, and was found to be one of the largest leftward asymmetries for surface area in addition to the pars opercularis of the inferior frontal gyrus (X.-Z. Kong et al., 2018). Lastly, the arcuate fasciculus has been found to be asymmetrical, as first identified byBüchel et al. (2004). As reviewed inOcklenburg et al. (2016), the left arcuate fasciculus has greater fractional anisotropy, fiber density, volume, and tract length than the right. Notably, apart from the Yakovlevian torque, each of the highlighted structures has an explicit tie to language function.

### 4.2 Evidence for the validity and reliability of NSAR

This measure was developed methodologically through the examination of ecological, convergent, and external validity, as well as a stable estimate analysis, test–retest reliability, and potential task effects (see sections 2.6 and 2.7; the Supplementary Methods). Notably, language task laterality appears to have a positive relationship with language network NSAR, suggesting that there is a degree of concordance between this resting-state measure of laterality and a task-based measure of laterality. Furthermore, estimates from this surface area approach to lateralization appear to converge with a different functional connectivity-based method (the autonomy index). This result supports the idea that NSAR is capturing lateralization in a way that is valid while being distinct from the autonomy index in how it is derived. Unlike the autonomy index, the formula for NSAR does not normalize for brain size or deal in the minutiae of individual functional connections. Rather, NSAR is calculated based on a network’s surface area. Additionally, potential relationships between network NSAR values and two cognitive measures were investigated in an analysis of external validity. Interestingly, a relationship between the laterality of the Visual-B, Language, Dorsal Attention-A, Control-C, and Default-C networks and language ability was identified. Similarly, others have identified a link between language function and left hemisphere lateralization during language production (Groen et al., 2012), and between the lateralization of functional networks and their associated cognitive abilities (Gotts et al., 2013).

Reliability analyses indicated that NSAR is stable within individuals, even after just 5 min of resting-state fMRI data. Interestingly, networks with the greatest reliabilities included the visual and somatomotor networks. This is in keeping withR. Kong et al. (2019), who found that sensorimotor networks exhibited lower intersubject functional connectivity variability than association networks. Since NSAR is indirectly based on an individual’s functional connectivity profiles, this result is unexpected.

In addition to the quantity of data available per participant, we also examined the effect of data type (task vs. rest) on NSAR estimates within individuals. While within-task type reliability was high, we found that there was indeed a task effect such that resting-state fMRI and task fMRI did not yield identical parcellations and NSAR estimates within individuals. This finding supports the hypothesis that resting-state fMRI can be thought of as another arbitrary task state (Buckner et al., 2013). Yet, the “task” of resting-state fMRI can result in greater variability in functional connectivity compared with that resulting from task fMRI, perhaps resulting from mind wandering (Elton & Gao, 2015). And when predicting individual traits, task-based models outperform rest-based models, with this difference likely reflecting the “unconstrained nature” of the resting state (Greene et al., 2018). Since NSAR estimates are derived from individual parcellations which are in turn generated from individual functional connectivity profiles, it stands to reason that connectivity differences resulting from task type could trickle down to differences in NSAR estimates.

### 4.3 The identification of eight reliably lateralized networks

Following the methodological development of NSAR, we reliably identified eight lateralized networks across three datasets: Visual-B, Language, Dorsal Attention-A, Salience/Ventral Attention-A, Control-B, Control-C, Default-C, and Limbic-B. While a ninth lateralized network was reliably identified (Control-A), this network was discarded from further analysis due to very poor reliability. Previously, several of these networks have been established as lateralized, particularly those associated with language and visuospatial attention processing.

#### 4.3.1 The Dorsal Attention-A network exhibited the greatest left lateralization

Previously, left-lateralized networks have included the language, frontoparietal control, and default networks. More specifically, evidence for the lateralization of the language network has been derived from a variety of methods including the Wada test (Desmond et al., 1995;Wada & Rasmussen, 1960), lesion cases (Broca, 1861;K. Wernicke, 1995), task fMRI (Elin et al., 2022;Fedorenko, Duncan, et al., 2012;Fedorenko et al., 2010,^2011^;Fedorenko, McDermott, et al., 2012;Lipkin et al., 2022;Malik-Moraleda et al., 2022;Olulade et al., 2020;Scott et al., 2017;Wilson et al., 2017), and resting-state fMRI (Braga et al., 2020;Labache et al., 2020;Zhu et al., 2014), among others. Using NSAR, we also identified the language network as being strongly left lateralized. However, unlike a prior comparative study (Braga et al., 2020), which examined lateralization in the language, salience, default, and frontoparietal networks (but not a dorsal attention network), we did not find that the language network was the most left-lateralized network. Instead, we identified the Dorsal Attention-A network as being the most left lateralized. Unlike the ventral attention network, the dorsal attention network has been previously identified as a bilateral network (Fox et al., 2006; for review seeMengotti et al., 2020). This was the case for the Dorsal Attention-B network, which was not a significantly lateralized network across the three datasets. However, there is evidence for a left-lateralized dorsal attention network across both left- and right-handed individuals, stemming from a within-individual network variants approach (see fig. 7 panel C ofPerez et al., 2023). Additionally, it could be that a finer-grained parcellation deconstructs the dorsal attention network into one bilateral and one lateralized network, similar to previous within-individual work on the default network (Braga & Buckner, 2017;DiNicola et al., 2020).

#### 4.3.2 Replication of right-lateralized attention, control, and limbic networks

This is not the first study to identify the ventral attention, control, and limbic networks as being lateralized. Abundant evidence exists for the right lateralization of visuospatial/ventral attention, stemming from task fMRI (Beume et al., 2015;Cai et al., 2013;Jansen et al., 2004;Shulman et al., 2010;Siman-Tov et al., 2007;Umarova et al., 2010;J. Wang et al., 2016;Zago et al., 2016,^2017^), resting-state fMRI (Braga et al., 2020;D. Wang et al., 2014), hemispatial neglect cases (Corbetta & Shulman, 2011), and others (for review, seeMengotti et al., 2020). Interestingly, we identified the Salience/Ventral Attention-A but not the Salience/Ventral Attention-B network as being right lateralized. Once more, this may be due to the network resolution selected (*k*= 17), which may have split the canonical ventral attention network into a bilateral and a right-lateralized network.

While this study successfully replicated right-lateralized control networks (Control-B and Control-C), a left-lateralized control network was not identified. Previously,D. Wang et al. (2014)found evidence for a dually lateralized frontoparietal control network using the autonomy index. It was suggested that this control network acted as a coupler between the two hemispheres to increase efficiency while simultaneously supporting within-hemisphere processes. This was also evidenced bySpreng et al. (2013), which found that the frontoparietal control network exhibits distinct connectivity patterns with the default and attention networks in response to varying task requirements. Similarly, using a seed-based analysis,Braga et al. (2020)confirmed the presence of both left-lateralized and right-lateralized frontoparietal control networks. Collectively, these results point to control networks differentially executing cognitive processes within the left and right hemispheres.

Finally, the most right-lateralized network identified using NSAR was the Limbic-B network, a network that occupies cortical real estate associated with emotion (Olson et al., 2007;Pehrs et al., 2017;Sonkusare et al., 2020). Historically, emotion processing has been identified as being lateralized, perhaps beginning with lesion cases (Gainotti, 2019;Hughlings-Jackson, 1878;Luys, 1879). Later work suggested that specific aspects of emotion were lateralized, including the right lateralization of emotion recognition, the right lateralization of emotional control and expression, the right lateralization of negative emotions, and the left lateralization of positive emotions (Silberman & Weingartner, 1986). Contemporarily, it has been suggested that a hemispheric functional-equivalence hypothesis would better explain emotion neuroimaging results, such that emotion results from networks that are interrelated and may have different patterns of lateralization (for review, seePalomero-Gallagher & Amunts, 2022). This perspective emphasizes the intricate and interconnected nature of emotion-related neural processes, particularly those patterns of lateralization that emerge from internetwork relationships. Interestingly, the Limbic-B network appears to be at the center of our main results regarding lateralization relationships between networks.

### 4.4 Support for the dependent hypothesis of network lateralization

Beyond identifying networks with the greatest lateralization, we sought to understand how network lateralization was related between networks. Framing this investigation, a 2019 review described relationships between lateralized brain networks in terms of functional complementarity, or the degree of specialization minimizing functional overlap and redundancy for a pair of networks (Vingerhoets, 2019). Borrowing from the author’s ecological differentiation metaphor, just as species may specialize and fill different niches, facilitating coexistence among species, brain networks may also operate in a complementary fashion through the use of distinct computational processes and neural locations. Conversely, species (and brain networks) which do not specialize are functionally redundant and face increased competition. Crucially, this dynamic is such that high complementarity characterizes brain networks with low redundancy and competition, while low complementarity describes brain networks with high redundancy and competition. To explore this further, we hypothesized that having one highly lateralized network corresponds with increased lateralization in other networks within an individual, and that this pattern of covariation would systematically occur across individuals (the dependent hypothesis). Interestingly, we identified lateralized network relationships exhibiting high and low complementarity occurring systematically across three different datasets.

#### 4.4.1 High complementarity network relationships

Using correlation matrices, we found support for the dependent hypothesis in networks lateralized to contralateral hemispheres and exhibiting high complementarity. A negative relationship was found between the right-lateralized Limbic-B network and the left-lateralized Dorsal Attention-A network. Such a relationship is indicative of covariation, since negative NSAR values indicate left hemisphere lateralization, so greater lateralization of the left-lateralized Dorsal Attention-A network (negative NSAR values) was associated with greater lateralization of the right-lateralized Limbic-B network (positive NSAR values). Similarly, an additional negative relationship was identified between the right-lateralized Limbic-B network and the left-lateralized Default-C network. These relationships were systematic across individuals spanning three datasets, suggesting that there may be a population benefit to this configuration of lateralization. Interestingly, while others have suggested that the relationship between linguistic and spatial processing networks may be characterized by high complementarity as well (Vingerhoets, 2019), we did not find evidence for this relationship in the present study.

#### 4.4.2 Low complementarity network relationships

Beyond the high complementarity relationships, using correlation matrices we also identified a dependent relationship for networks lateralized to the same hemisphere exhibiting low complementarity. Since NSAR is derived based on surface area and the selected network parcellation method has a winner-takes-all approach, all cortical surface area for each individual is accounted for and networks lateralized to the same hemisphere are in competition with one another for cortical real estate. Thus, it is not surprising that two networks lateralized to the same hemisphere might have a negative relationship, such as that between the left-lateralized Dorsal Attention-A and Language networks identified in the present study. This relationship is such that as the lateralization for the Dorsal Attention-A network increases, the lateralization of the language network decreases within individuals or vice versa, and this pattern was consistent across individuals from three datasets. Remarkably, two other examples of this low complementarity relationship have previously been identified and both involve language or linguistic processing. First, one group found evidence for the “colateralization” of language and praxis networks on both the individual and group levels (Vingerhoets et al., 2013). A similar “colateralization” relationship was identified for language and arithmetic regions (Pinel & Dehaene, 2010). In the latter study, it was suggested that “colateralization” might hint at the developmental effects of learning linguistic symbols on the organization of the arithmetic network. An additional hypothesis for this type of complementary relationship suggests that networks composed of overlapping nodes in the same hemisphere may be so similar that sharing proximate space is biologically less costly than the generation of a separate redundant network in the opposite hemisphere (Vingerhoets, 2019).

#### 4.4.3 Further evidence of the dependent hypothesis

Additional support for the dependent hypothesis was found with the EFA and CFA structures across the three datasets. While we did not replicate the four-factor model fromLiu et al. (2009), we did extract two factors for the HCP-Discovery dataset, which were then fitted in the HCP-Replication and HCPD datasets. Across these factor analyses, significant positive and negative loadings were found within each factor structure, suggesting that left- and right-lateralized networks work within a system level higher than the network.

#### 4.4.4 Characteristics of lateralized brain network organization

Together, the present evidence accumulated from the correlation matrices and EFA and CFA structures points to three overlapping features of organization for lateralized networks: complementarity, plasticity, and hierarchy. Beyond identifying which networks are lateralized, the present study evidences a configuration in which there are trade-offs in redundancy and competition. Rather than operating in isolation, lateralized networks appear to function in a larger system where their organization is interdependent. Given the zero-sum nature of a surface area-based approach, one might argue that this interconnectedness is an artifact. However, evidence from prior task fMRI lateralization research is in support of interconnectedness (Pinel & Dehaene, 2010;Vingerhoets et al., 2013). Similarly, the presented results demonstrating that network lateralization strength is related between networks suggest a degree of plasticity and adaptability in the brain’s functional organization. This potential developmental influence was hinted at with the “colateralization” of language and arithmetic; however, longitudinal models are needed to verify this speculation. Lastly, the present results describe a hierarchy of lateralized brain networks. This is most clearly demonstrated with the EFA and CFA structures, which were composed of both positive and negative factor loadings, suggesting that these lateralized networks are not isolated but rather part of a larger system. This hierarchical organization of lateralized networks implies a mosaic of interaction and dependency within the broader brain architecture.

### 4.5 Limitations and future directions

One limitation to this work is that while functional connectivity may be constrained in part by anatomical connectivity, it is not necessarily dictated by anatomical connectivity. Several pieces of evidence point to this conclusion: functional connectivity is modulated by task (Shirer et al., 2012), recent experience (Lewis et al., 2009), caffeine (Laumann et al., 2015), and sleepiness (Tagliazucchi & Laufs, 2014); and is dynamic within a person over time (Hutchison et al., 2013). Furthermore, underlying brain geometry models of spontaneous neural activity appear to be more accurate and parsimonious than those derived from anatomical connectivity (Pang et al., 2023). Hence, NSAR as a connectivity and surface area-based measure is more reflective of functional rather than anatomical lateralization. As a result, future studies might benefit from exploring theCoutanche et al. (2023)method, which employs a surface-fingerprinting technique and multivariate laterality index for computing functional lateralization, offering a potentially complementary approach to NSAR in assessing functional lateralization.

In this study, individual parcellations were generated using theR. Kong et al. (2019)MS-HBM algorithm. However, improved versions of this algorithm have since been published (R. Kong et al., 2021;Yan et al., 2023), which account for parcel distributions, spatial contiguity, local gradients, and homotopy (or the lack thereof in Schaefer parcels). Thus, future investigations using NSAR might consider implementing an updated individual parcellation algorithm. Moreover, it would be valuable for future studies to explore lateralization in developmental and clinical populations to address questions regarding the developmental timeline of network lateralization and the potential disruptions in network lateralization observed in specific neurodevelopmental conditions such as autism or schizophrenia.

## 5 Conclusions

The present study investigated hemispheric asymmetries in the human brain, focusing on 17 functional networks. This was accomplished by implementing a surface area-based metric of lateralization, for which validity and reliability were examined. Following methodological development, we addressed two main questions: (1) which networks exhibit the greatest hemispheric asymmetries and (2) how does lateralization in one network relate to the lateralization of other networks? We found that the Language, Dorsal Attention-A, and Default-C networks were significantly left lateralized while the Visual-B, Salience/Ventral Attention-A, Control-B, Control-C, and Limbic-B networks were significantly right lateralized. Additionally, using correlation matrices and EFA and CFA models to understand how lateralization is related between networks, we found general support for a dependent relationship between left- and right-lateralized networks. Within individuals, greater left lateralization in a particular network (such as the Dorsal Attention-A or Default-C networks) was associated with greater right lateralization in a particular network (such as the Limbic-B network). This pattern of lateralization appears to occur systematically across individuals, suggesting that lateralization follows a covariation paradigm. Further work is needed to understand how these findings may differ in developmental and clinical populations.

## Supplementary Material

Supplementary Material

## Data Availability

Apart from the HCPD dataset, the data reported on in the present study can be accessed publicly online (HCP:https://db.humanconnectome.org/; NSD:http://naturalscenesdataset.org/). The HCPD dataset is hosted through the NIMH Data Archive (NDA), through which access can be requested. Preprocessing and individual parcellation pipeline codes are available through the CBIG repository on GitHub athttps://github.com/ThomasYeoLab/CBIG. Scripts used to implement the processing pipelines and perform statistical analyses are available on GitHub athttps://github.com/Nielsen-Brain-and-Behavior-Lab/NSAR2023.
