# Supplementary material for: Evidence for a compensatory relationship between left- and right-lateralized brain networks
Source: Imaging Neurosci (Camb). 2025 Jan 29;3:imag_a_00437. doi: 10.1162/imag_a_00437 (PMC12319987; doi:10.1162/imag_a_00437)
Supplement: Supplementary Material [file imag_a_00437-supp.pdf]

Evidence for a Compensatory Relationship between Left- and Right-Lateralized Brain Networks

Supplementary Materials

1 Supplementary Methods

This section provides additional information on estimating the validity and reliability of NSAR. Additional figures and tables referenced in the main text are then presented.

1.1 Establishing the Validity of NSAR

The ecological validity of Language network laterality was initially explored, specifically investigating how Language network NSAR scores (derived from resting-state data) predict real-world behaviors such as laterality from a language task. The Language network was selected since it has previously been established as a highly lateralized network. HCP subjects with all four runs of resting-state data and the minimally preprocessed Story-Math task contrast were selected ($N = 221$). This Story-Math task was used as a proxy for a language task (specifically, language comprehension), as has been done previously (Labache et al., 2023; Lin et al., 2022; Wang et al., 2023). Participant *t*-statistic contrast maps were converted to fsaverage6 resolution using wb_command functions *cifti-separate* and *metric-resample*, masked using a language task fMRI atlas (LanA atlas) derived from a large sample ($N = 804$; Lipkin et al., 2022), and then thresholded to the top 10% of vertices. We chose this threshold rather than a fixed *t*-value in order to account for individual differences in the strength of BOLD signal responses attributable to individual differences arising from trait or state factors (Lipkin et al., 2022). A simple laterality metric was then calculated for each contrast map: the number of right hemisphere vertices minus the number of left hemisphere vertices divided by the sum of the left and right hemisphere vertices. A Spearman rank correlation was then used to compare language task laterality against the NSAR value for the Language network. This and all other statistical analyses took place in R 4.2.0 (R Core Team, 2022).

Convergent validity was also examined through a comparison of the NSAR against a measure of specialization: the autonomy index (Wang et al., 2014). The autonomy index approaches specialization from a functional connectivity perspective and is known to reliably estimate specialization across neurotypical and clinical samples (Mueller et al., 2015; Sun et al., 2022; Wang et al., 2014). First, individual functional connectivity matrices were calculated for each resting-state run and then averaged across runs within an individual at the fsaverage6 resolution in MATLAB R2018b (MATLAB, 2018). From here the autonomy index was computed as follows: for each seed ROI obtained from a functional connectivity matrix, the degree of within-hemisphere connectivity and cross-hemisphere connectivity were computed by summing the number of vertices correlated to the seed in the ipsilateral hemisphere and in the contralateral hemisphere. These vertex counts are then normalized by the total number of vertices in the corresponding hemisphere, thus accounting for a potential brain size asymmetry between the two hemispheres. Finally, AI is calculated as the difference between normalized within- and cross-hemisphere connectivity as follows:

$$AI = N_i/H_i - N_c/H_c$$

where N_i and N_c are the number of vertices correlated to the seed ROI (using a threshold of |0.25|) in the ipsilateral hemisphere and contralateral hemisphere, respectively. H_i and H_c are the total number of vertices in the ipsilateral and contralateral hemisphere, respectively. To compute the specialization of each functional network, the AI was averaged within the boundary of each network on an individual basis. Subjects from the HCP dataset with all four runs available (N = 232) were selected for this analysis of validity and all four runs from each individual were used to compute the autonomy index. Since the autonomy index is computed separately for each hemisphere, a positive value is indicative of specialization (i.e., a left-specialized network may

have positive autonomy index values, as may a right-specialized network). A Spearman's rank correlation coefficient was then used to compare the autonomy index and NSAR on three right-lateralized networks (Limbic-B, Visual-B, and Ventral Attention-A) and three left-lateralized networks (Language, Dorsal Attention-A, and Control-B) determined *a priori*. In order to correct for multiple comparisons, a Bonferroni-corrected alpha level of 0.008 was used (derived from dividing 0.05 by six networks).

External validity was next examined through a comparison of NSAR values from eight networks (Visual-B, Language, Dorsal Attention-A, Salience/Ventral Attention-A, Control-B, Control-C, Default-C, and Limbic-B) against two measures from the Cognition Battery of the National Institutes of Health Toolbox (Gershon et al., 2013): the Oral Reading Recognition Test (ORRT; Gershon et al., 2014) and the Flanker Inhibitory Control and Attention Test (adapted from Rueda et al., 2004). The ORRT was selected as a measure of language and the Flanker as a measure of executive control (inhibitory control, specifically) and visuospatial attention. Among all available cognitive assessments, we selected those that have be shown to engage cognitive domains lateralized to both the right (assessing attention via the Flanker test) and left (evaluating language through the ORRT) hemispheres. Each cognitive measure has been highly validated (Heaton et al., 2014; Ott et al., 2022; Zelazo et al., 2014). To facilitate the comparison of NSAR against these cognitive measures, a Canonical Correlation Analysis (CCA) was implemented using HCP subjects with all four resting-state runs available ($N = 232$). In preparation for the CCA, the age- and sex-adjusted values from the two cognitive measures were evaluated in pairwise plots, which were followed by the Doornik-Hansen multivariate test for normality (*DH.test* function from the mvnTest package; (version 1.1.0; Doornik & Hansen, 2008; Pya et al., 2016). The CCA was chosen for its ability to robustly estimate relationships between sets of

variables (Marek et al., 2022) and was conducted using the *cc* function from the CCA package in R (version 1.2.2; González & Déjean, 2023). CCA feature weights were Haufe-transformed (Haufe et al., 2014) in order to provide a more realistic perspective of feature contributions considering the covariance structure of the data. Haufe-transformations are also known to increase the interpretability and reliability of feature weights (Chen, Ooi, et al., 2022; Chen, Tam, et al., 2022; Tian & Zalesky, 2021)

1.2 Establishing the Reliability of NSAR

Reliability analyses sought to address three questions: 1) How much data is needed to obtain a stable estimate of NSAR, 2) What is the test-retest reliability of NSAR, and 3) Is there a task effect on NSAR estimation?

1.2.1 Stable Estimate Analysis

Given that MRI scanning is costly, rendering it comparatively rare to have highly sampled individuals, it is important to understand how much data is needed to reliably estimate lateralization and assess the credibility of our results. To address this concern, we analyzed HCP participants with all four runs of resting-state data available ($N = 232$). Following preprocessing, the first and third scans from each participant were set aside to compose 30 minutes of independent data. Next, the second and fourth scans were each split into three five-minute segments. Runs were split in MATLAB R2018b (MATLAB, 2018) using native MATLAB functions as well as the FreeSurfer functions *MRIread* and *MRIwrite*. The MS-HBM pipeline was then used to generate individual parcellations from 5, 10, 15, 20, 25, and 30 minutes of data from the segmented scans. The MS-HBM pipeline was also used to generate separate individual parcellations from 30 minutes of independent data. Of note, the reliability of the MS-HBM pipeline has been examined previously (see Kong et al. (2019) Figure 3B and Supplementary

Figure S10C). The NSAR was then calculated for each iteration (5, 10, 15, etc. minutes) and the independent 30 minutes of data. An intraclass correlation between the NSAR from each iteration parcellation and the independent 30 minutes parcellation was assessed within each subject. Similarly, an intraclass correlation between the NSAR from each iteration parcellation and the independent 30 minutes parcellation was assessed for each network. For the NSAR and parcellation stable estimate analyses, the standard guidelines from Koo & Li (2016) regarding intraclass correlation values were implemented, with values less than 0.5 indicating poor reliability, values between 0.5 and 0.75 indicating moderate reliability, values between 0.75 and 0.9 indicating good reliability, and values greater than 0.9 indicating excellent reliability (based on a 95% confidence interval).

Similarly, a stable estimate analysis was performed to determine the reliability of the parcellations themselves. A dice coefficient (Dice, 1945; Sorenson, 1948) was calculated in order to identify parcellation label overlap between the parcellations resulting from iteration (5, 10, 15, etc. minutes) and the parcellation resulting from the independent 30 minutes of data. The dice coefficient is calculated as follows:

$$\text{Dice} = \frac{2|X \cap Y|}{|X|+|Y|}$$

where $X \cap Y$ represents the number of vertices with the same network labels in the same positions across the iteration parcellation and the independent 30 minutes parcellation. The denominator represents the total number of vertices with a given network label across both the iteration parcellation and the independent 30 minutes parcellation. Dice coefficients were also estimated for each network within each participant between each increment of data and the 30 independent minutes of data, and then an average dice coefficient for each network was computed for each increment of data.

1.2.2 Test-Retest Reliability Analysis

The purpose of the test-retest reliability analysis is to measure the reliability of NSAR in a simpler fashion than the stable estimate analysis. For this analysis, the first two and second two runs from HCP participants with all four runs available were used to generate separate individual parcellations from which NSAR will be calculated. Outliers were fenced on a network basis to an upper limit of the third quartile plus 1.5 multiplied by the interquartile range, and a lower limit of the first quartile minus 1.5 multiplied by the interquartile range. An intraclass correlation coefficient was calculated comparing the NSAR from the first half of the data with the NSAR from the second half for three right-lateralized networks (Limbic-B, Visual-B, and Salience/Ventral Attention-A) and three left-lateralized networks (Language, Dorsal Attention-A, and Control-B) determined *a priori*.

1.2.3 Task Effects Analysis

In the case that a large quantity of data is needed to derive a reliable estimate of lateralization, one might consider including task data in addition to any resting-state data in order to increase the amount of available data per participant. However, in this situation it would be prudent to know if task data provides the same or similar estimates as those from resting-state data. To address this concern, the NSD dataset was selected since it has a large quantity of both resting-state and task-based fMRI data per participant. Following preprocessing, a minimum of 12 resting-state runs were available for each participant, so the first 12 available resting-state runs and the first 12 available task runs were utilized (resting-state and task runs were of the same duration). Individual parcellations were then generated based on various combinations of runs within task type: even-numbered runs, odd-numbered runs, the first half of runs, the second half of runs, and two random selections of runs (without replacement). A dice coefficient was

then computed to compare parcellation label overlap within task (e.g., between even and odd-numbered resting-state runs) and between tasks (e.g., between odd-numbered runs from resting-state and task runs). This comparison procedure was repeated for the NSAR intraclass correlation coefficient. Due to the non-normal nature of such a small dataset, comparisons between the task and rest parcellation dice coefficients and NSAR intraclass correlations were formally made using paired Wilcoxon Signed Rank tests (R Core Team, 2011; Wilcoxon, 1945).

2 Additional Tables and Figures

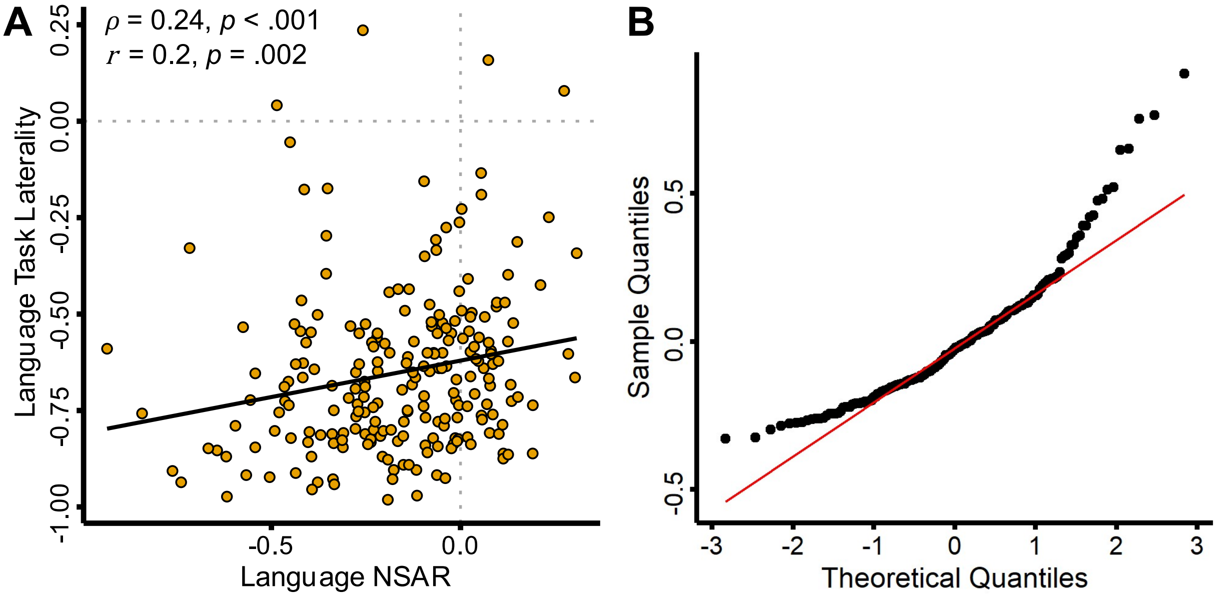

Figure S1. Language task laterality and Language network NSAR. Panel A depicts a positive relationship between NSAR for the Language network and language task laterality in a subset of the HCP dataset (*N* = 221). Across each measure of laterality, a negative value denotes left-hemisphere lateralization while a positive value indicates right-hemisphere lateralization. Panel B depicts a Quantile-Quantile plot, which displays the residuals for the modeled relationship between language task laterality and Language network NSAR displayed in Panel A.

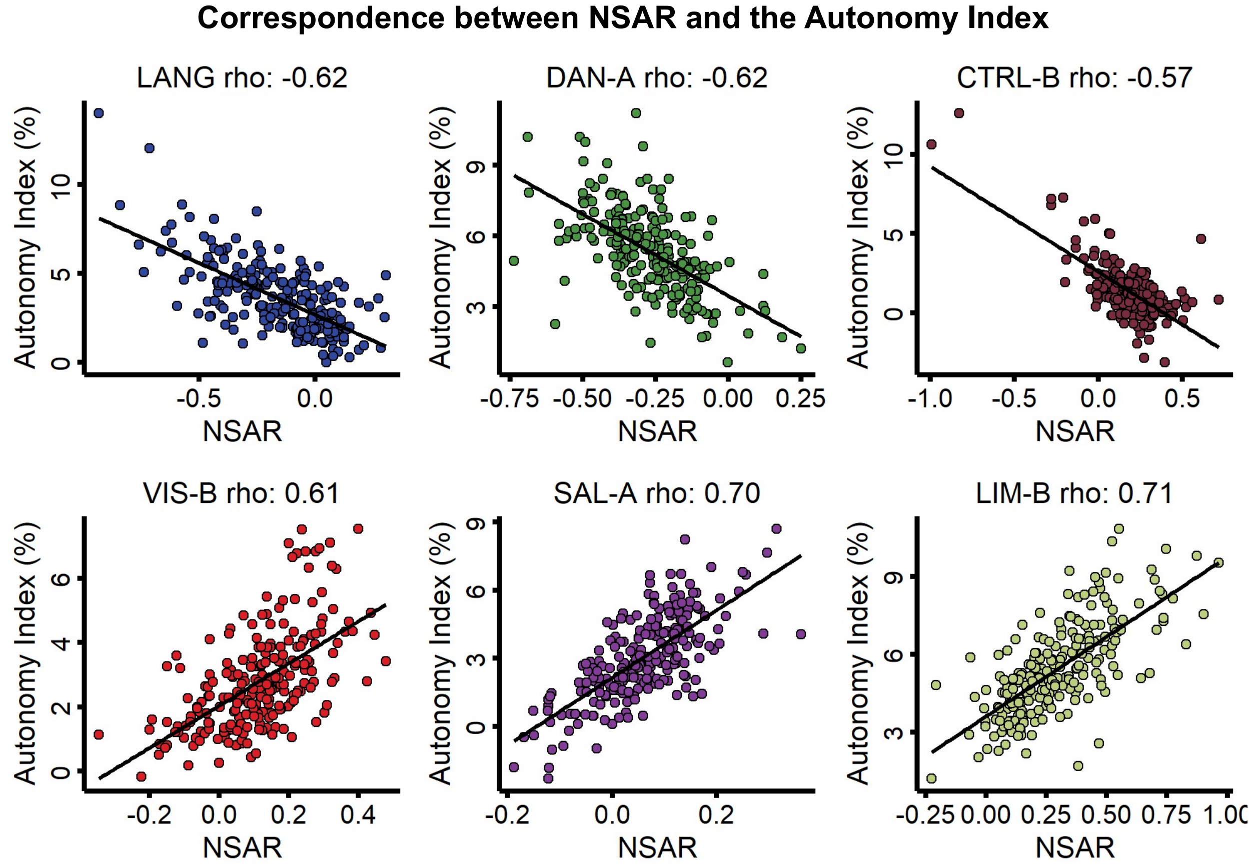

Figure S2. Evidence for convergent validity between the autonomy index and NSAR in a subset of HCP dataset. The top row depicts the relationships between the autonomy index and NSAR for three left-lateralized networks (Language, Dorsal Attention-A, and Control-B; Spearman rank correlation ρ = -0.57 - -0.62). The bottom row depicts the relationships between the autonomy index and NSAR for three right-lateralized networks (Visual-B, Ventral Attention-A, and Limbic-B; Spearman rank correlation ρ = 0.61 - 0.71). For each scatterplot, the line of best fit was generated using the *lm* function (no covariates) and each circle represents an individual.

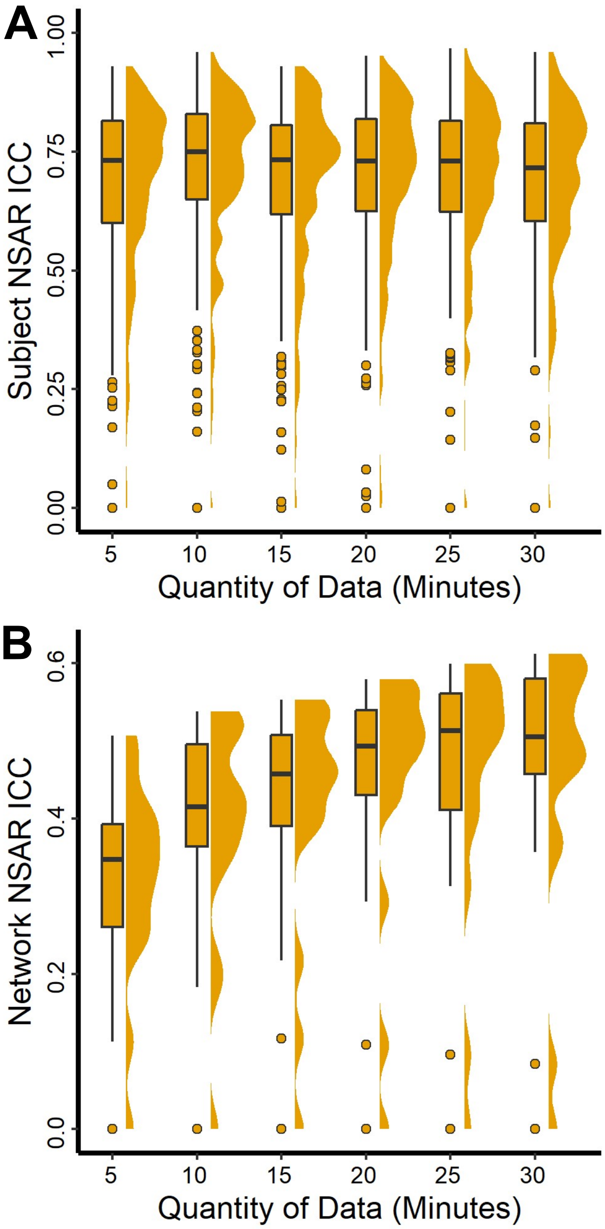

Figure S3. Evidence for reliable estimates of NSAR in the HCP dataset. Panel A depicts the intraclass correlation coefficient calculated for each subject's 17 NSAR values for each time increment (5, 10, 15 … 30 minutes) and the subject's 17 NSAR values from 30 independent minutes of data. Panel B depicts the intraclass correlation coefficient calculated for each network's mean NSAR value between the 30 independent minutes of data and each increment of data. The distribution of intraclass correlation coefficients is shown for the 17 networks. Specific network intraclass correlation coefficients are displayed in Figure S4.

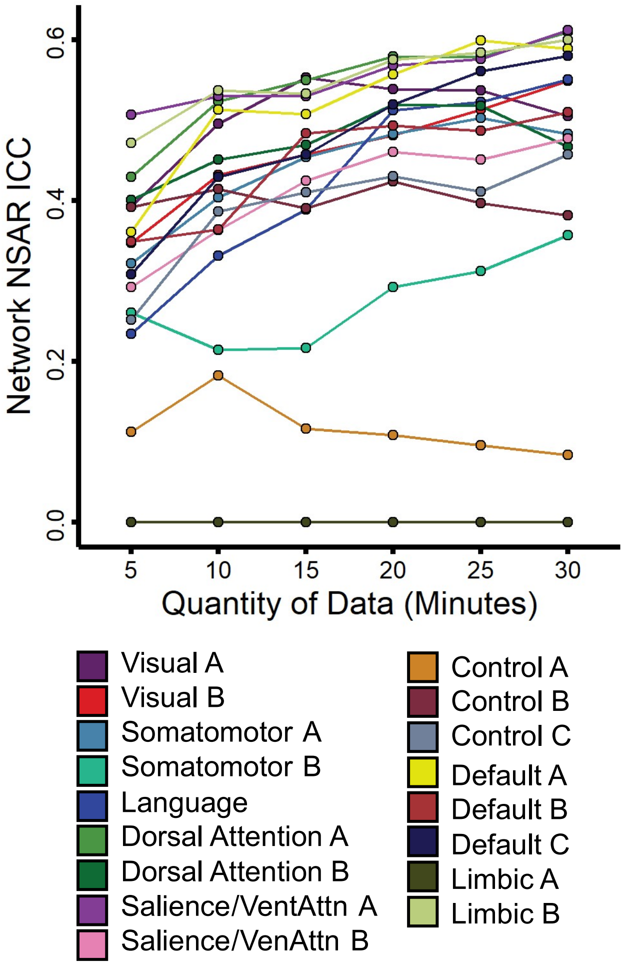

Figure S4. NSAR network reliability. Depicted is the intraclass correlation coefficient calculated for each network's mean NSAR value between the 30 independent minutes of data and each increment of data.

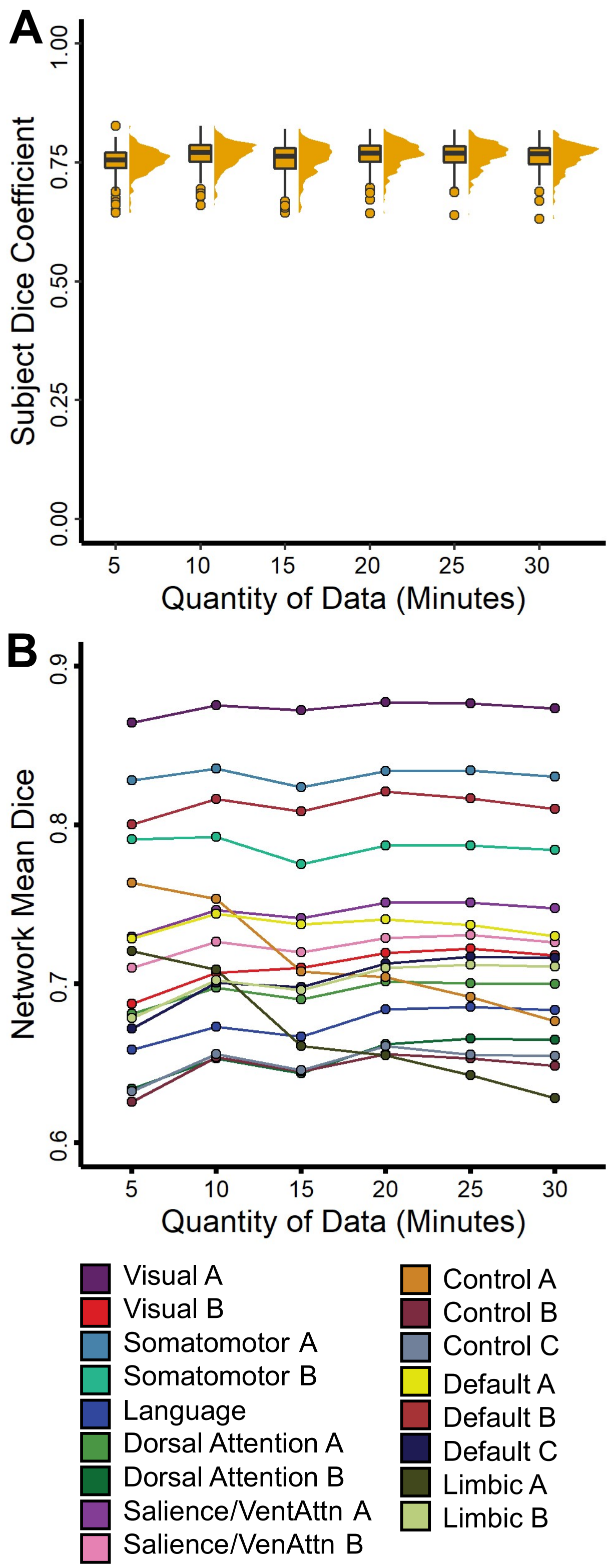

Figure S5. Parcellation overlap estimates in a subset of participants from the HCP dataset (*N* = 232). Panel A depicts the subject dice coefficients calculated for the individual parcellations between each

increment of data (5, 10, 15, … 30 minutes) and 30 independent minutes of data. Dice coefficient calculations are detailed in the Supplementary Methods. Comparable subject-level network assignment overlap analyses were conducted in the Midnight Scan Club dataset (see Gordon et al., 2017 Figure 2 Panel B), finding that dice coefficients began at ~0.4 - 0.6 and plateaued at ~0.6 - 0.8. Panel B depicts the subject dice coefficients calculated for the individual parcellations between each increment of data (5, 10, 15, … 30 minutes) and 30 independent minutes of data averaged within each network. Comparable test-retest findings for parcellation overlap were described in Kong et al. (2019) Figures S10 Panel C and S11 Panel C, for which the somatomotor and visual networks exhibited the greatest intra-subject reproducibility.

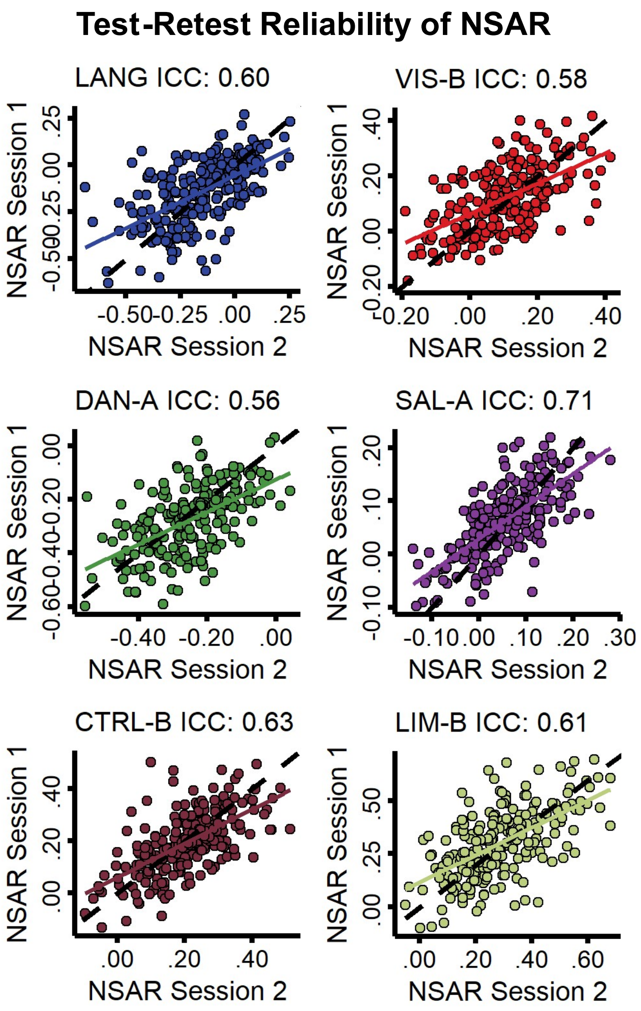

Figure S6. Test-retest reliability of NSAR values for left- and right-lateralized networks in 232 HCP subjects. Left-lateralized networks (left column) included Language, Dorsal Attention-A, and Control-B. Right-lateralized networks (right column) included Visual-B, Salience/Ventral Attention-A, and Limbic-B. In each plot, a circle represents a subject.

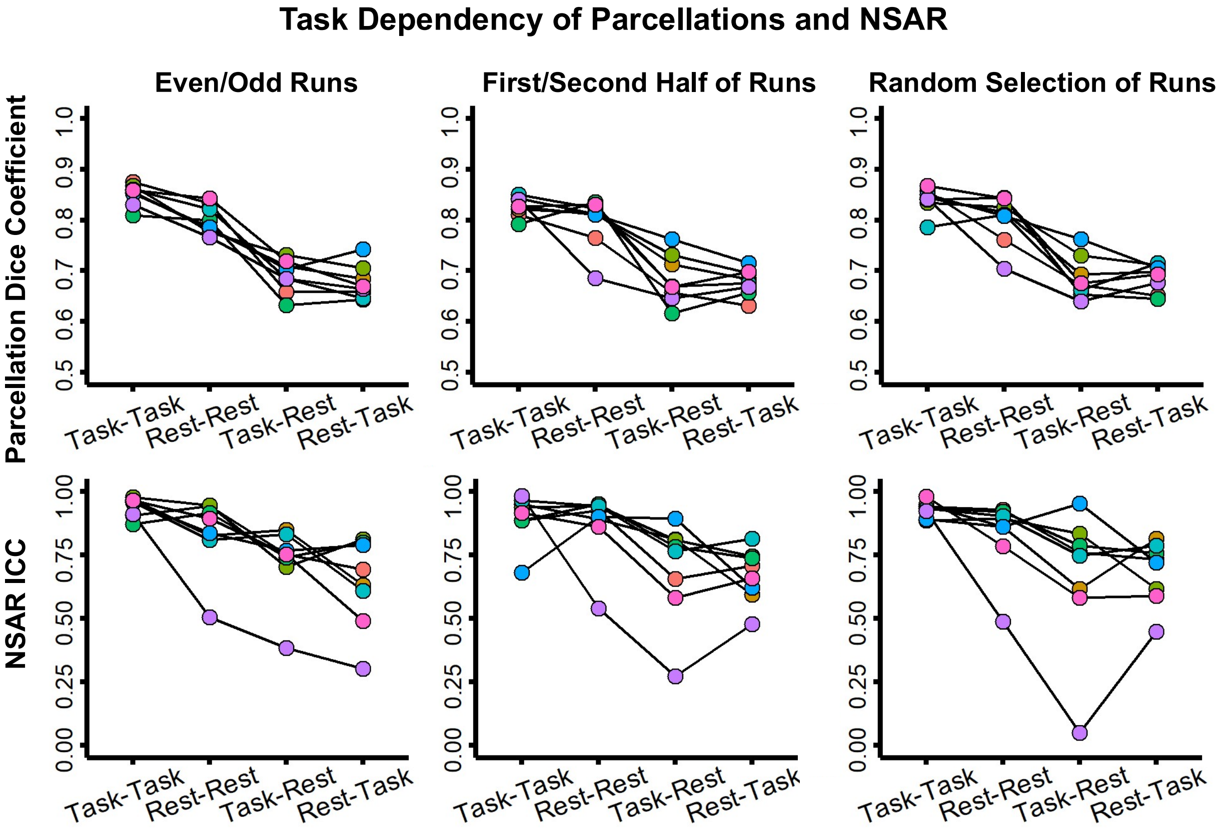

Figure S7. Task dependency of individual parcellations and NSAR in the NSD dataset. Depicted in the top row are the dice coefficients for the individual parcellations between 30-minute increments of resting-state or task fMRI data. Regardless of how the data were split (even- versus odd-numbered runs, the first half versus the second half, or a random selection without replacement), a task effect was found. Depicted in the second row are the NSAR intraclass correlation coefficients computed in individuals across networks. In each plot, circles connected by a line represent an individual.

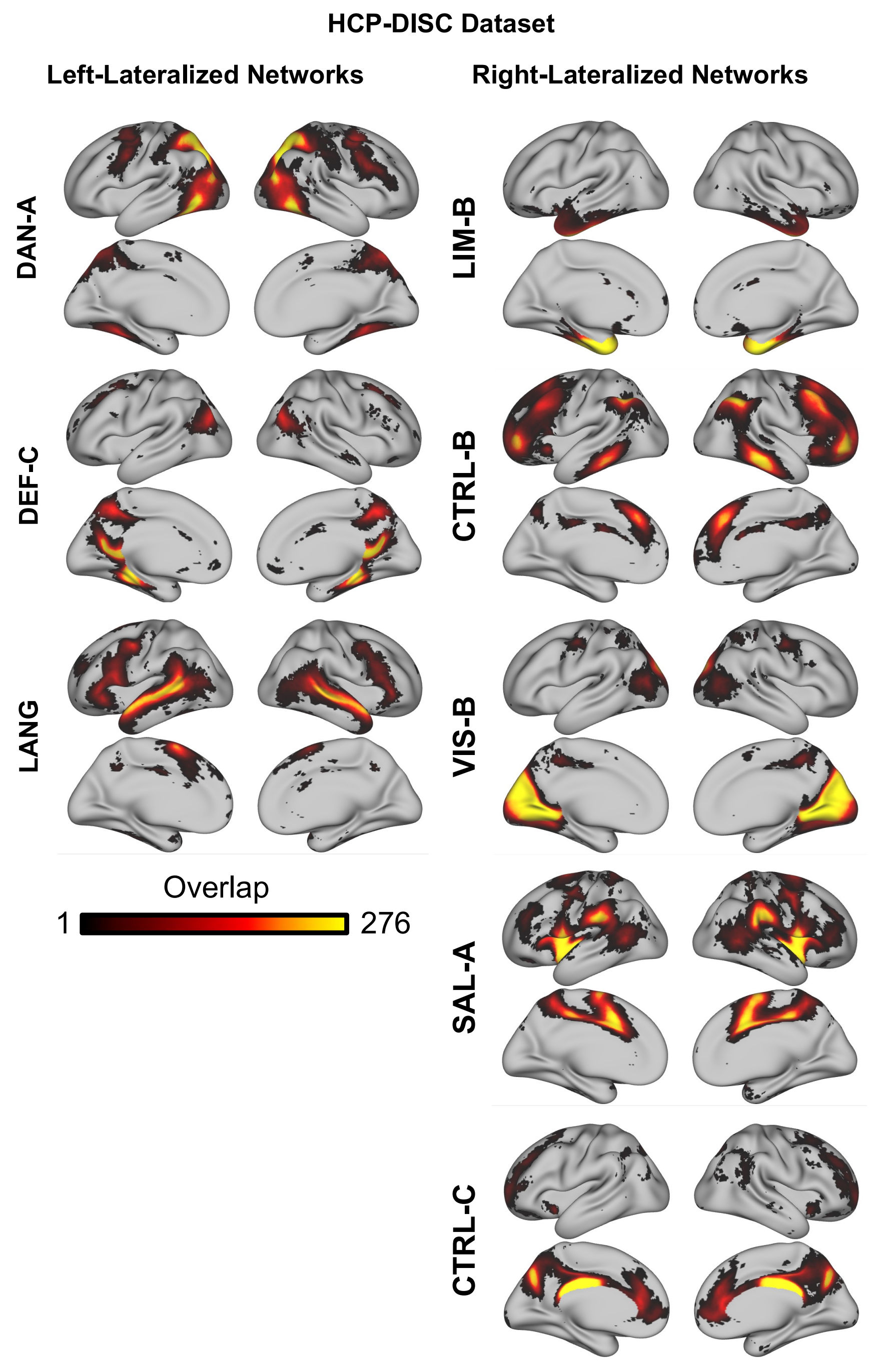

Figure S8. Overlap of network estimates derived from the individual parcellations in the HCP-Discovery dataset (*N* = 276). Each network map displays the overlap in for that network on the fsaverage6 surface for 276 participants using the estimates from the 17-network Multi-Session Hierarchical Bayesian Modeling algorithm. The left column displays the three significantly left-lateralized networks (Dorsal Attention-A, Default-C, and Language) while the right column displays the five significantly right-lateralized networks (Limbic-B, Control-B, Visual-B, Salience/Ventral Attention-A, and Control-C).

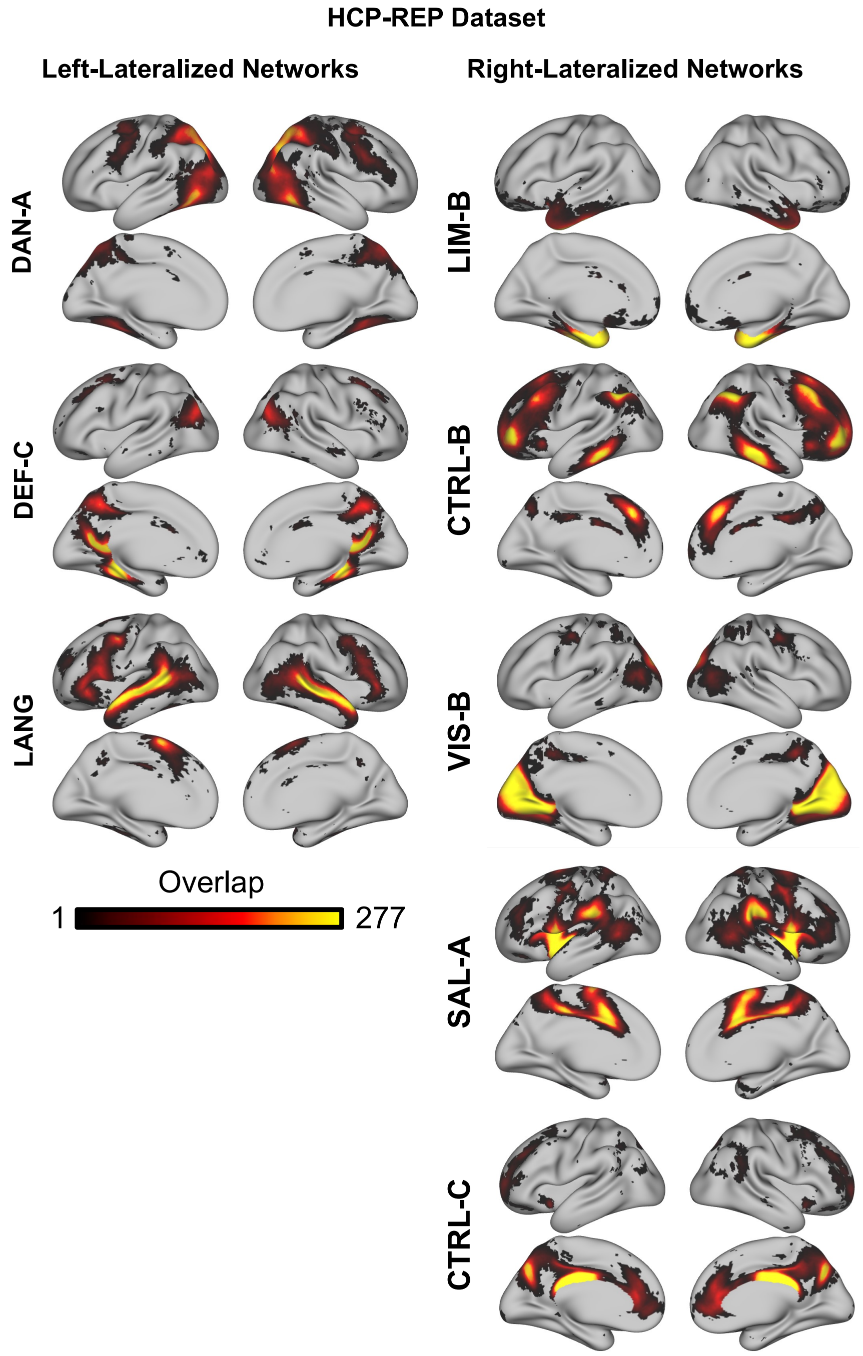

Figure S9. Overlap of network estimates derived from the individual parcellations in the HCP-Replication dataset (*N* = 277). Each network map displays the overlap in for that network on the fsaverage6 surface for 277 participants using the estimates from the 17-network Multi-Session Hierarchical Bayesian Modeling algorithm. The left column displays the three significantly left-lateralized networks (Dorsal

Attention-A, Default-C, and Language) while the right column displays the five significantly right-lateralized networks (Limbic-B, Control-B, Visual-B, Salience/Ventral Attention-A, and Control-C).

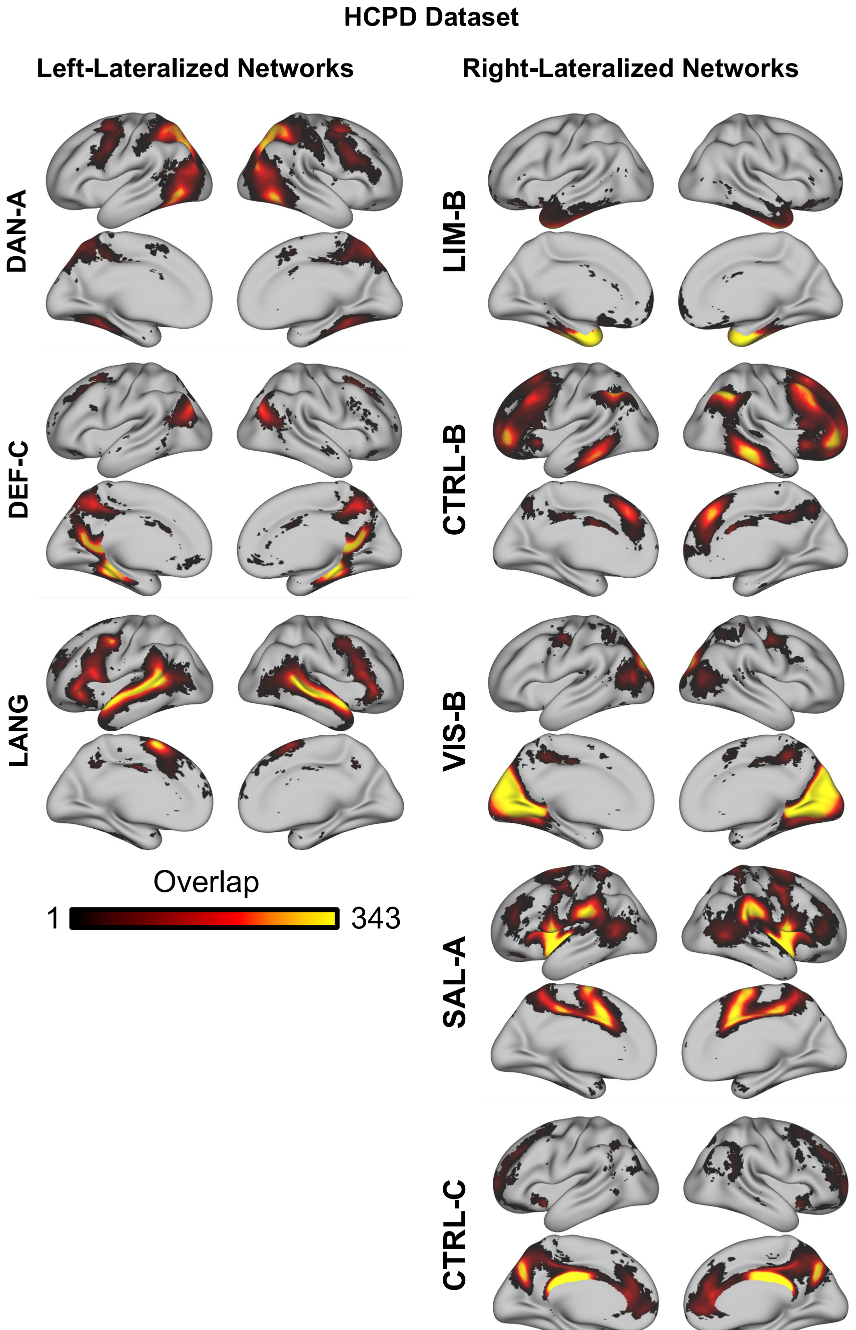

Figure S10. Overlap of network estimates derived from the individual parcellations in the HCPD dataset (*N* = 343). Each network map displays the overlap in for that network on the fsaverage6 surface for 343 participants using the estimates from the 17-network Multi-Session Hierarchical Bayesian Modeling

algorithm. The left column displays the three significantly left-lateralized networks (Dorsal Attention-A, Default-C, and Language) while the right column displays the five significantly right-lateralized networks (Limbic-B, Control-B, Visual-B, Salience/Ventral Attention-A, and Control-C).

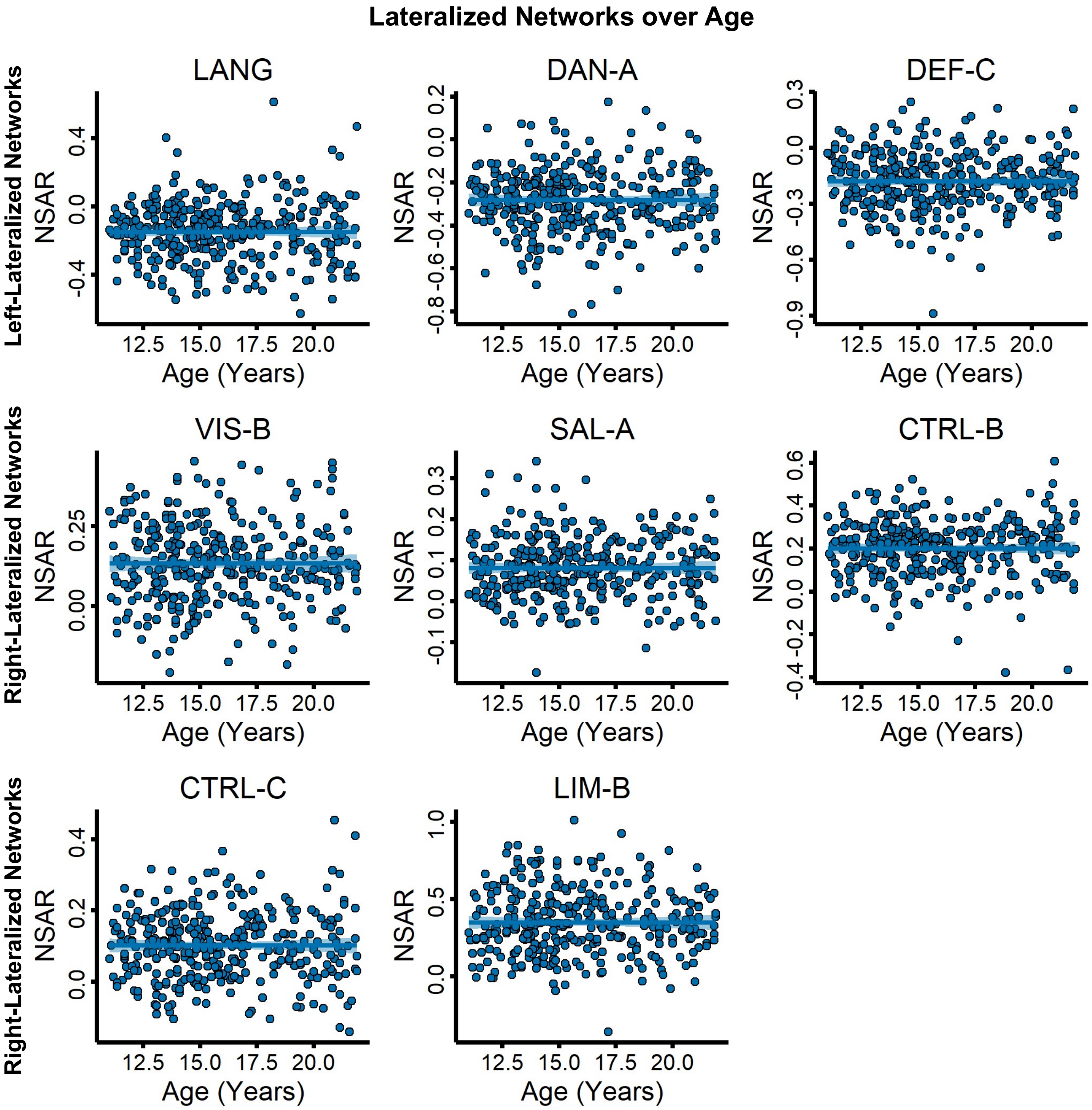

Figure S11. Eight lateralized networks over age in HCPD participants (*N* = 343). The top row depicts three left-lateralized networks: Language, Dorsal Attention-A, and Default-C. The bottom two rows depict five right-lateralized networks: Visual-B, Salience/Ventral Attention-A, Control-B, Control-C, and Limbic-B.

In each plot, NSAR were adjusted by regressing out the effects of mean-centered age, mean-centered mean framewise displacement, and sex using the following formula: $NSAR_{adjusted} = NSAR_{raw}$ — [β_1(mean-centered age_{raw} – mean of mean-centered age_{raw}) + β_2(mean-centered FD_{raw} – mean of mean-centered FD_{raw}) + β_3(sex_{raw} – mean sex_{raw}) + β_4($handedness_{raw}$ – mean $handedness_{raw}$)]. Age was not a significant covariate for any of the eight lateralized networks.

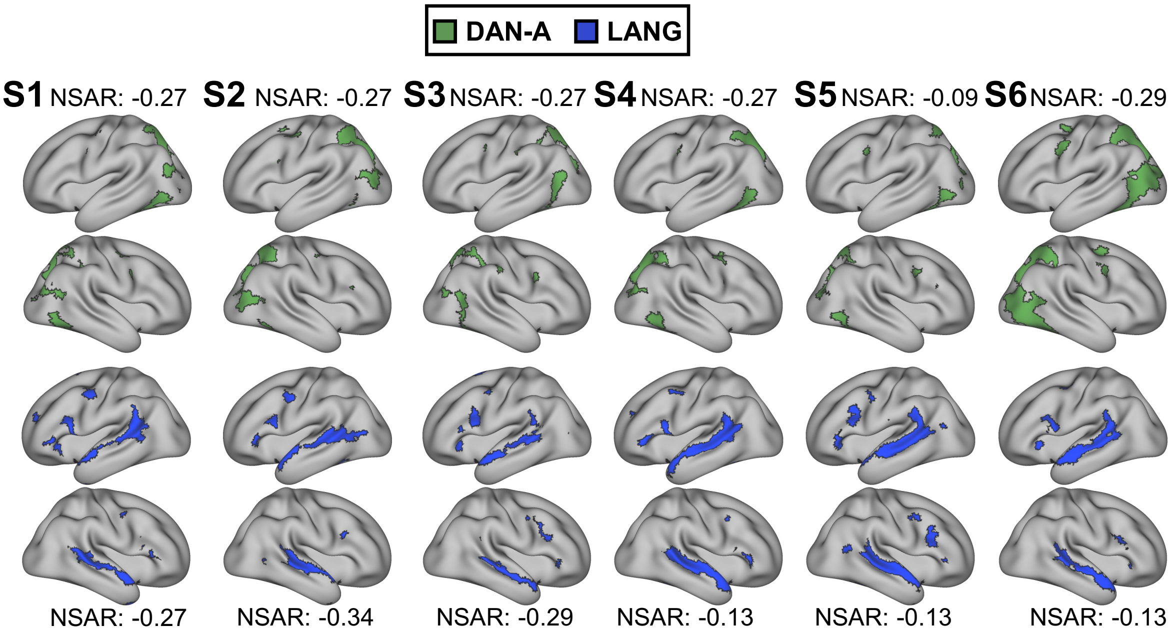

Figure S12. Functional neuroanatomy of the Dorsal Attention-A (DAN-A) and Language (LANG) networks. Subjects S1-S3 had median NSAR values for the DAN-A network (depicted in green) and subjects S4-S6 had median NSAR values for the LANG network (depicted in blue). Subjects were selected across the HCP-Discovery, HCP-Replication, and HCPD datasets. The individual network parcellations were overlaid on an fsaverage6 midthickness surface for display purposes.

Table S1

Canonical Correlation Analysis Results for Dimension 1in a Subset of the HCP Dataset (N = 232)

	Standardized Canonical Coefficient	Haufe-Transformed Weight	Correlation with Canonical Variate*

Cognitive Variables			
Language (ORRT)	-0.99	-0.45	**-0.32**
Attention (Flanker)	0.39	2.22	0.07
Lateralized Networks			
Visual-B	-0.33	-0.05	**-0.13**
Language	0.39	0.13	**0.2**
Dorsal Attention-A	-0.54	-0.07	**-0.17**
Salience/Ventral Attention-A	-0.18	-0.03	-0.1
Control-B	0.11	-0.01	-0.02
Control-C	0.48	0.04	**0.13**
Default-C	0.37	0.05	0.12
Limbic-B	-0.17	-0.03	-0.05

*Bolded values were significant at the $p < .05$ level following multiple comparison corrections.

Table S2

Identifying Specialized Networks Using Multiple Regressions in the HCP-Discovery (N = 276), HCP-Replication (N = 277), and HCPD (N = 343) Datasets

Network Intercept	Dataset	β	*SE*	*t*	*p*
**Visual-A**					
	HCP-DISC	0.02	0.01	3.63	< .001
	HCP-REP	0.01	0.01	2.79	.006
	HCPD	0.01	0.00	1.54	.13
**Visual-B**					
	HCP-DISC	0.07	0.02	3.95	< .001
	HCP-REP	0.11	0.02	6.26	< .001
	HCPD	0.11	0.01	8.18	< .001
Somatomotor-A					

	HCP-DISC	0.02	0.01	2.92	.004
	HCP-REP	0.01	0.01	1.41	.16
	HCPD	0.01	0.01	1.22	.22
Somatomotor-B					
	HCP-DISC	-0.03	0.01	-3.35	< .001
	HCP-REP	-0.03	0.01	-2.85	.005
	HCPD	-0.01	0.01	-3.42	< .001
**Language**					
	HCP-DISC	-0.15	0.03	-4.71	< .001
	HCP-REP	-0.12	0.03	-4.13	< .001
	HCPD	-0.19	0.02	-8.11	< .001
**Dorsal Attention-A**					
	HCP-DISC	-0.25	0.02	-12.05	< .001
	HCP-REP	-0.31	0.02	-15.37	< .001
	HCPD	-0.22	0.02	-13.74	< .001
Dorsal Attention-B					
	HCP-DISC	-0.02	0.02	-1.24	.21
	HCP-REP	-0.03	0.02	-1.47	.14
	HCPD	0.01	0.01	0.87	.38
**Salience/VenAttn-A**					
	HCP-DISC	0.05	0.01	4.48	< .001
	HCP-REP	0.07	0.01	6.05	< .001
	HCPD	0.05	0.01	6.35	< .001
Salience/VenAttn-B					
	HCP-DISC	-0.02	0.01	-1.59	.11
	HCP-REP	-0.03	0.01	-2.56	.01
	HCPD	-0.03	0.01	-3.52	< .001
**Control-A**					

	HCP-DISC	-0.07	0.02	-3.7	< .001
	HCP-REP	-0.11	0.02	-6.51	< .001
	HCPD	-0.05	0.01	-6.52	< .001
**Control-B**					
	HCP-DISC	0.19	0.02	7.95	< .001
	HCP-REP	0.18	0.02	8.09	< .001
	HCPD	0.14	0.01	9.93	< .001
**Control-C**					
	HCP-DISC	0.09	0.02	6.11	< .001
	HCP-REP	0.09	0.01	7.16	< .001
	HCPD	0.09	0.01	9.53	< .001
Default-A					
	HCP-DISC	-0.02	0.02	-1.01	.31
	HCP-REP	-0.02	0.02	-1.52	.13
	HCPD	-0.01	0.01	-1.04	.3
Default-B					
	HCP-DISC	0.01	0.01	1.04	.29
	HCP-REP	0.02	0.01	2.13	.03
	HCPD	0.01	0.01	0.87	.39
**Default-C**					
	HCP-DISC	-0.21	0.02	-10.56	< .001
	HCP-REP	-0.17	0.02	-9.37	< .001
	HCPD	-0.15	0.02	-8.83	< .001
Limbic-A					
	HCP-DISC	0.06	0.01	4.4	< .001
	HCP-REP	0.09	0.01	6.3	< .001
	HCPD	0.01	0.01	0.91	.37
**Limbic-B**					

HCP-DISC	0.25	0.03	8.93	< .001
HCP-REP	0.32	0.03	11.89	< .001
HCPD	0.28	0.02	12.21	< .001

Note: Coefficients and *p*-values for the intercept are shown. None of the covariates (mean-centered age, mean-centered framewise displacement, handedness, and sex) were consistently significant across the three datasets for any of the networks. Networks with reliably significant (Bonferroni-corrected alpha level of .003) intercepts are bolded.

Table S3

Left-lateralized Network Comparisons

Network Comparison	Dataset	β	*SE*	*t*	*p*
Language					
Dorsal Attention-A					
	HCP-DISC	-0.11	0.02	-6.98	< .001
	HCP-REP	-0.11	0.02	-6.73	< .001
	HCPD	-0.12	0.01	-9.69	< .001
Default-C					
	HCP-DISC	0.09	0.01	7.94	< .001
	HCP-REP	0.1	0.01	8.63	< .001
	HCPD	0.11	0.01	9.09	< .001
Dorsal Attention-A					
Default-C					
	HCP-DISC	-0.01	0.02	-0.93	.36
	HCP-REP	-0.00	0.02	-0.31	.76
	HCPD	-.02	0.01	-1.29	.19

Note: Comparisons consist of multiple regressions, which included a network variable with two levels (the two networks under comparison), mean-centered age, sex, handedness, and mean-centered mean framewise displacement.

Table S4

Right-lateralized Network Comparisons

Network Comparison	Dataset	*β*	*SE*	*t*	*p*
Visual-B					
Salience/VenAttn-A					
	HCP-DISC	-0.04	0.01	-4.56	< .001
	HCP-REP	-0.05	0.01	-5.04	< .001
	HCPD	-0.05	0.01	-6.69	< .001
Control-B					
	HCP-DISC	0.09	0.02	5.69	< .001
	HCP-REP	0.08	0.01	6.19	< .001
	HCPD	0.05	0.01	5.47	< .001
Control-C					
	HCP-DISC	-0.01	0.01	-1.03	.3
	HCP-REP	-0.01	0.01	-1.27	.2
	HCPD	-0.01	0.01	-1.56	.12
Limbic-B					
	HCP-DISC	0.08	0.02	4.21	< .001
	HCP-REP	0.18	0.01	12.89	< .001
	HCPD	0.18	0.01	13.46	< .001
Salience/VenAttn-A					
Control-B					
	HCP-DISC	0.06	0.01	4.32	< .001
	HCP-REP	0.12	0.01	11.18	< .001
	HCPD	0.11	0.01	13.1	< .001
Control-C					
	HCP-DISC	0.03	0.01	3.71	< .001

		HCP-REP	0.03	0.01	4.29	< .001
		HCPD	0.04	0.07	6.04	< .001
	Limbic-B					
		HCP-DISC	0.21	0.01	16.23	< .001
		HCP-REP	0.22	0.01	17.81	< .001
		HCPD	0.23	0.01	19.23	< .001
Control-B						
	Control-C					
		HCP-DISC	-0.09	0.01	-6.97	< .001
		HCP-REP	-0.09	0.01	-7.78	< .001
		HCPD	-0.07	0.01	-7.59	< .001
	Limbic-B					
		HCP-DISC	0.09	0.02	5.89	< .001
		HCP-REP	0.1	0.02	6.74	< .001
		HCPD	0.12	0.01	9.32	< .001
Control-C						
	Limbic-B					
		HCP-DISC	0.18	0.01	13.04	< .001
		HCP-REP	0.19	0.01	14.69	< .001
		HCPD	0.19	0.01	15.35	< .001

Note: Comparisons consist of multiple regressions, which included a network variable with two levels (the two networks under comparison), mean-centered age, sex, handedness, and mean-centered mean framewise displacement.

3 Supplementary References

Chen, J., Ooi, L. Q. R., Li, J., Asplund, C. L., Eickhoff, S. B., Bzdok, D., Holmes, A., & Yeo, B. (2022). There is no fundamental trade-off between prediction accuracy and feature importance reliability. *BioRxiv*.

Chen, J., Tam, A., Kebets, V., Orban, C., Ooi, L. Q. R., Asplund, C. L., Marek, S., Dosenbach, N. U. F., Eickhoff, S. B., Bzdok, D., Holmes, A. J., & Yeo, B. T. T. (2022). Shared and unique brain network features predict cognitive, personality, and mental health scores in the ABCD study. *Nature Communications*, *13*(1), Article 1. https://doi.org/10.1038/s41467-022-29766-8

Dice, L. R. (1945). Measures of the amount of ecologic association between species. *Ecology*, *26*(3), 297–302. https://doi.org/10.2307/1932409

Doornik, J. A., & Hansen, H. (2008). An omnibus test for univariate and multivariate normality. *Oxford Bulletin of Economics and Statistics*, *70*, 927–939.

Gershon, R. C., Cook, K. F., Mungas, D., Manly, J. J., Slotkin, J., Beaumont, J. L., & Weintraub, S. (2014). Language Measures of the NIH Toolbox Cognition Battery. *Journal of the International Neuropsychological Society : JINS*, *20*(6), 642–651. https://doi.org/10.1017/S1355617714000411

Gershon, R. C., Wagster, M. V., Hendrie, H. C., Fox, N. A., Cook, K. F., & Nowinski, C. J. (2013). NIH Toolbox for Assessment of Neurological and Behavioral Function. *Neurology*, *80*(11 Supplement 3), S2–S6. https://doi.org/10.1212/WNL.0b013e3182872e5f

González, I., & Déjean, S. (2023). *CCA: Canonical Correlation Analysis* (Version 1.2.2) [Computer software]. https://cran.r-project.org/web/packages/CCA/index.html

Gordon, E. M., Laumann, T. O., Gilmore, A. W., Newbold, D. J., Greene, D. J., Berg, J. J., Ortega, M., Hoyt-Drazen, C., Gratton, C., Sun, H., Hampton, J. M., Coalson, R. S., Nguyen, A. L., McDermott, K. B., Shimony, J. S., Snyder, A. Z., Schlaggar, B. L., Petersen, S. E., Nelson, S. M., & Dosenbach, N. U. F. (2017). Precision functional mapping of individual human brains. *Neuron*, *95*(4), Article 4. https://doi.org/10.1016/j.neuron.2017.07.011

Haufe, S., Meinecke, F., Görgen, K., Dähne, S., Haynes, J.-D., Blankertz, B., & Bießmann, F. (2014). On the interpretation of weight vectors of linear models in multivariate neuroimaging. *NeuroImage*, *87*, 96–110. https://doi.org/10.1016/j.neuroimage.2013.10.067

Heaton, R. K., Akshoomoff, N., Tulsky, D., Mungas, D., Weintraub, S., Dikmen, S., Beaumont, J., Casaletto, K. B., Conway, K., Slotkin, J., & Gershon, R. (2014). Reliability and Validity of Composite Scores from the NIH Toolbox Cognition Battery in Adults. *Journal of the International Neuropsychological Society : JINS*, *20*(6), 588–598. https://doi.org/10.1017/S1355617714000241

Kong, R., Li, J., Orban, C., Sabuncu, M. R., Liu, H., Schaefer, A., Sun, N., Zuo, X.-N., Holmes, A. J., Eickhoff, S. B., & Yeo, B. T. T. (2019). Spatial topography of individual-specific cortical networks predicts human cognition, personality, and emotion. *Cerebral Cortex*, *29*(6), Article 6. https://doi.org/10.1093/cercor/bhy123

Koo, T. K., & Li, M. Y. (2016). A Guideline of Selecting and Reporting Intraclass Correlation Coefficients for Reliability Research. *Journal of Chiropractic Medicine*, *15*(2), 155–163. https://doi.org/10.1016/j.jcm.2016.02.012

Labache, L., Ge, T., Yeo, B. T., & Holmes, A. J. (2023). Language network lateralization is reflected throughout the macroscale functional organization of cortex. *Nature Communications*, *14*(1), 3405.

Lin, L., Chang, D., Song, D., Li, Y., & Wang, Z. (2022). Lower resting brain entropy is associated with stronger task activation and deactivation. *NeuroImage*, *249*, 118875.

Lipkin, B., Tuckute, G., Affourtit, J., Small, H., Mineroff, Z., Kean, H., Jouravlev, O., Rakocevic, L., Pritchett, B., Siegelman, M., Hoeflin, C., Pongos, A., Blank, I. A., Struhl, M. K., Ivanova, A., Shannon, S., Sathe, A., Hoffmann, M., Nieto-Castañón, A., & Fedorenko, E. (2022). *LanA (Language Atlas): A probabilistic atlas for the language network based on fMRI data from >800 individuals* (p. 2022.03.06.483177). bioRxiv. https://doi.org/10.1101/2022.03.06.483177

Marek, S., Tervo-Clemmens, B., Calabro, F. J., Montez, D. F., Kay, B. P., Hatoum, A. S., Donohue, M. R., Foran, W., Miller, R. L., Hendrickson, T. J., Malone, S. M., Kandala, S., Feczko, E., Miranda-Dominguez, O., Graham, A. M., Earl, E. A., Perrone, A. J., Cordova, M., Doyle, O., … Dosenbach, N. U. F. (2022). Reproducible brain-wide association studies require thousands of individuals. *Nature*, *603*(7902), Article 7902. https://doi.org/10.1038/s41586-022-04492-9

MATLAB. (2018). *9.5.0.944444 (R2018b)*. The MathWorks Inc.

Mueller, S., Wang, D., Pan, R., Holt, D. J., & Liu, H. (2015). Abnormalities in hemispheric specialization of caudate nucleus connectivity in schizophrenia. *JAMA Psychiatry*, *72*(6), 552–560. https://doi.org/10.1001/jamapsychiatry.2014.3176

Ott, L. R., Schantell, M., Willett, M. P., Johnson, H. J., Eastman, J. A., Okelberry, H. J., Wilson, T. W., Taylor, B. K., & May, P. E. (2022). Construct validity of the NIH toolbox

cognitive domains: A comparison with conventional neuropsychological assessments. *Neuropsychology*, *36*(5), 468–481. https://doi.org/10.1037/neu0000813

Pya, N., Voinov, V., Makarov, R., & Voinov, Y. (2016). *mvnTest: Goodness of Fit Tests for Multivariate Normality* (Version 1.1-0) [Computer software]. https://cran.r-project.org/web/packages/mvnTest/index.html

R Core Team. (2011). *Wilcoxon rank sum and signed rank tests*. R Documentation.[Online]. Available: http://stat. ethz. ch/R-manual/R ….

R Core Team. (2022). *R: A Language and Environment for Statistical Computing*. R Foundation for Statistical Computing. https://www.R-project.org/

Rueda, M. R., Fan, J., McCandliss, B. D., Halparin, J. D., Gruber, D. B., Lercari, L. P., & Posner, M. I. (2004). Development of attentional networks in childhood. *Neuropsychologia*, *42*(8), 1029–1040. https://doi.org/10.1016/j.neuropsychologia.2003.12.012

Sorenson, T. (1948). A method of establishing groups of equal amplitude in plant sociology based on similarity of species content, and its application to analyses of the vegetation on Danish commons. *K Dan Vidensk Selsk Biol Skr*, *5*, 1–34.

Sun, J., Gao, X., Hua, Q., Du, R., Liu, P., Liu, T., Yang, J., Qiu, B., Ji, G.-J., Hu, P., & Wang, K. (2022). Brain functional specialization and cooperation in Parkinson's disease. *Brain Imaging and Behavior*, *16*(2), 565–573. https://doi.org/10.1007/s11682-021-00526-4

Tian, Y., & Zalesky, A. (2021). Machine learning prediction of cognition from functional connectivity: Are feature weights reliable? *NeuroImage*, *245*, 118648. https://doi.org/10.1016/j.neuroimage.2021.118648

Wang, D., Buckner, R. L., & Liu, H. (2014). Functional specialization in the human brain estimated by intrinsic hemispheric interaction. *Journal of Neuroscience*, *34*(37), Article 37. https://doi.org/10.1523/JNEUROSCI.0787-14.2014

Wang, X., Krieger-Redwood, K., Zhang, M., Cui, Z., Wang, X., Karapanagiotidis, T., Du, Y., Leech, R., Bernhardt, B. C., Margulies, D. S., & others. (2023). Physical distance to sensory-motor landmarks predicts language function. *Cerebral Cortex*, *33*(8), 4305–4318.

Wilcoxon, F. (1945). Individual comparisons by ranking methods. *Biometrics Bulletin*, *1*(6), 80–83. https://doi.org/10.2307/3001968

Zelazo, P. D., Anderson, J. E., Richler, J., Wallner-Allen, K., Beaumont, J. L., Conway, K. P., Gershon, R., & Weintraub, S. (2014). NIH Toolbox Cognition Battery (CB): Validation of Executive Function Measures in Adults. *Journal of the International Neuropsychological Society*, *20*(6), 620–629. https://doi.org/10.1017/S1355617714000472
